# *Bacillus* Calmette–Guérin Treatment Changes the Tumor Microenvironment of Non-Muscle-Invasive Bladder Cancer

**DOI:** 10.3389/fonc.2022.842182

**Published:** 2022-03-03

**Authors:** Fei Su, Ming Liu, Wei Zhang, Min Tang, Jinsong Zhang, Hexin Li, Lihui Zou, Rui Zhang, Yudong Liu, Lin Li, Jie Ma, Yaqun Zhang, Meng Chen, Fei Xiao

**Affiliations:** ^1^Clinical Biobank, Beijing Hospital, National Center of Gerontology, Institute of Geriatric Medicine, Chinese Academy of Medical Sciences, Beijing, China; ^2^Department of Urology, Beijing Hospital, National Center of Gerontology, Institute of Geriatric Medicine, Chinese Academy of Medical Sciences, Beijing, China; ^3^Department of Pathology, Beijing Hospital, National Center of Gerontology, Institute of Geriatric Medicine, Chinese Academy of Medical Sciences, Beijing, China; ^4^Department of Oncology, Beijing Hospital, National Center of Gerontology, Institute of Geriatric Medicine, Chinese Academy of Medical Sciences, Beijing, China; ^5^The Key Laboratory of Geriatrics, Beijing Hospital, National Center of Gerontology, Institute of Geriatric Medicine, Chinese Academy of Medical Sciences, Beijing, China; ^6^National Center for Clinical Laboratories, Beijing Hospital, National Center of Gerontology, Institute of Geriatric Medicine, Chinese Academy of Medical Sciences, Beijing, China; ^7^Center for Biotherapy, Beijing Hospital, National Center of Gerontology, Institute of Geriatric Medicine, Chinese Academy of Medical Sciences, Beijing, China; ^8^State Key Lab of Molecular Oncology, National Cancer Center, Chinese Academy of Medical Sciences Cancer Hospital & Peking Union Medical College, Beijing, China; ^9^National Cancer Data Center, National Cancer Center, Chinese Academy of Medical Sciences Cancer Hospital & Peking Union Medical College, Beijing, China

**Keywords:** *Bacillus* Calmette–Guérin, non-muscle-invasive bladder cancer, immune checkpoint, tumor microenvironment, neoantigen

## Abstract

**Background:**

*Bacillus* Calmette–Guérin (BCG) is currently the most effective intravesical therapy for non-muscle-invasive bladder cancer (NMIBC) as it can prevent disease recurrence and progression and lower mortality. However, the response rates to BCG vary widely and are dependent on a multitude of factors.

**Methods:**

We performed a systematic discovery by analyzing the whole exome sequence, expression profile, and immune repertoire sequence of treatment-naive and 5-year time-serial relapsed tumors from 24 NMIBC patients.

**Results:**

BCG therapy showed bidirectional effects on tumor evolution and immune checkpoint landscape, along with a significant reduction of the percentage of neoantigen burden. In addition, a remarkable proportion of subclonal mutations were unique to the matched pre- or post-treatment tumors, suggesting the presence of BCG-induced and/or spatial heterogeneity. In the relapsed tumors, we identified and validated a shift in the mutational signatures in which mutations associated with aristolochic acid (AA) exposure were enriched, implying AA may be associated with tumor recurrence. Enhanced expressions of immune checkpoint regulation genes were found in the relapsed tumors, suggesting that the combination of immune checkpoint with BCG treatment may be an effective strategy to treat NMIBC. TCR sequencing revealed treatment-associated changes in the T-cell repertoire in the primary and relapsed tumors.

**Conclusion:**

Our results provide insight into the genomic and immune dynamics of tumor evolution with BCG treatment, suggest new mechanisms of BCG resistance, and inform the development of clinically relevant biomarkers and trials of potential immune checkpoint inhibitor combination therapies.

## Introduction

Bladder cancer is the ninth most common malignancy worldwide and the fifth most common cancer in Europe and the United States, with the highest incidence found in southern European countries (1). Of the estimated 80,500 patients diagnosed with bladder cancer in China each year, 70%–80% had non-muscle-invasive bladder cancer (NMIBC) (2, 3). Intravesical *Bacillus* Calmette–Guérin (BCG) instillation is the “gold standard” adjuvant treatment after transurethral resection of the bladder tumors in high-risk NMIBC patients. Although BCG immunotherapy has markedly improved the outcome of NMIBC, the response rates to BCG vary from 32.6% to 42.1%; in addition, the progression rates range from 9.5% to 13.4% and are dependent on a multitude of factors (4).

Although many studies have suggested that BCG immunotherapy elicits multistep processes involving immunologic responses, molecular biomarkers for the prediction of BCG response have not been applied clinically, and the dynamic changes of genomic profile during BCG treatment are still unknown. In particular, the extent to which bladder tumors adapt to immune environment disturbed by the live-attenuated *Mycobacterium bovis* remains unknown. In addition, whether and when to discontinue BCG and implement a more aggressive therapy is the hardest decision faced by urologists when managing high-risk NMIBC patients. According to previous studies (EORTC and CUETO bladder cohorts), clinicopathologic features, especially tumor stage, carcinoma *in situ* (CIS), and grade, are the most effective predictors of BCG response (5, 6). Accurate prediction of treatment response before BCG instillation will be particularly valuable for selecting an appropriate therapeutic modality. Blood biomarkers, such as lymphocyte, neutrophil, and platelet counts, reflect the balance of host inflammatory and immune status and are an established prognostic factor in bladder cancer (7). In addition, new targeted immunotherapies approved for many cancer types have shed new light on BCG-induced immune response, which may guide the development of specific therapies to effectively harness the immune system.

In our current study, we recruited 24 treatment-naive NMBIC patients, treated them with BCG, and followed them for more than 5 years. We performed whole-exome sequencing (WES), RNA sequencing, and immune repertoire sequencing (IR-seq) to analyze matched sets of primary, recurrent, and germline samples, with an attempt to address the fundamental question: How does BCG affect the tumor microenvironment (TME) between primary and relapsed bladder tumors?

## Results

### Selected Patient Characteristics and Genomic Landscape

From 2012 to 2014, 24 patients with intermediate- or high-risk NMIBC were recruited to receive BCG treatment (Immunobladder^®^; Japan BCG Laboratory, Japan). The selected clinical characteristics of patients and primary tumors were as follows: 41.7% (10/24) single tumors and 58.3% (14/24) multiple tumors, 54.2% (13/24) Ta and 45.8% (11/24) T1, and 41.7% (10/24) G2 and 58.3% (14/24) G3 (14/24), and none had prior intravesical chemotherapy or radiotherapy (**Table 1**). According to the study of Huang et al. (8), all patients received a 1-year scheduled BCG therapy: six weekly induction and three weekly repeated maintenance sessions. The median follow-up period after BCG therapy was 73.5 months [95% confidence interval (CI), 67.47–79.50 months]. Eleven patients had tumor recurrence, five of which were locally advanced (T2+). The 3- and 5-year recurrence-free survival (RFS) rates after BCG therapy were 58.3% (CI: 36.6%–77.9%) and 50% (CI: 29.1%–70.9%), respectively. Cystoscopy about every 3 months was done to check the tumor status. Additional details on the NMIBC cohort are summarized in **Table S1**.

**Table 1:** Patient and tumor characteristics.

Variable	BCG treatment
Age (years)	
50–59	7/24 (29.2%)
60–69	8/24 (33.3%)
70–79	9/24 (37.5%)
Gender	
Male	21/24 (87.5%)
Female	3/24 (12.5%)
No. of tumors	
Single	10/24 (41.7%)
Multiple	14/24 (58.3%)
Tumor size (d/cm)	
0–3	15/24 (62.5%)
≥3	9/24 (37.5%)
T category	
Ta	13/24 (54.2%)
T1	11/24 (45.8%)
Grade	
G2	10/24 (41.7%)
G3	14/24 (58.3%)
CIS	
Yes	5/24 (20.8%)
No	19/24 (79.2%)
EORTC recurrence score	
≤9 (intermediate risk)	22/24 (91.7%)
10–17 (high risk)	2/24 (8.3%)
EORTC progression score	
≤6 (intermediate risk)	10/24 (41.7%)
7–23 (high risk)	14/24 (58.3%)

**Figure 1:**
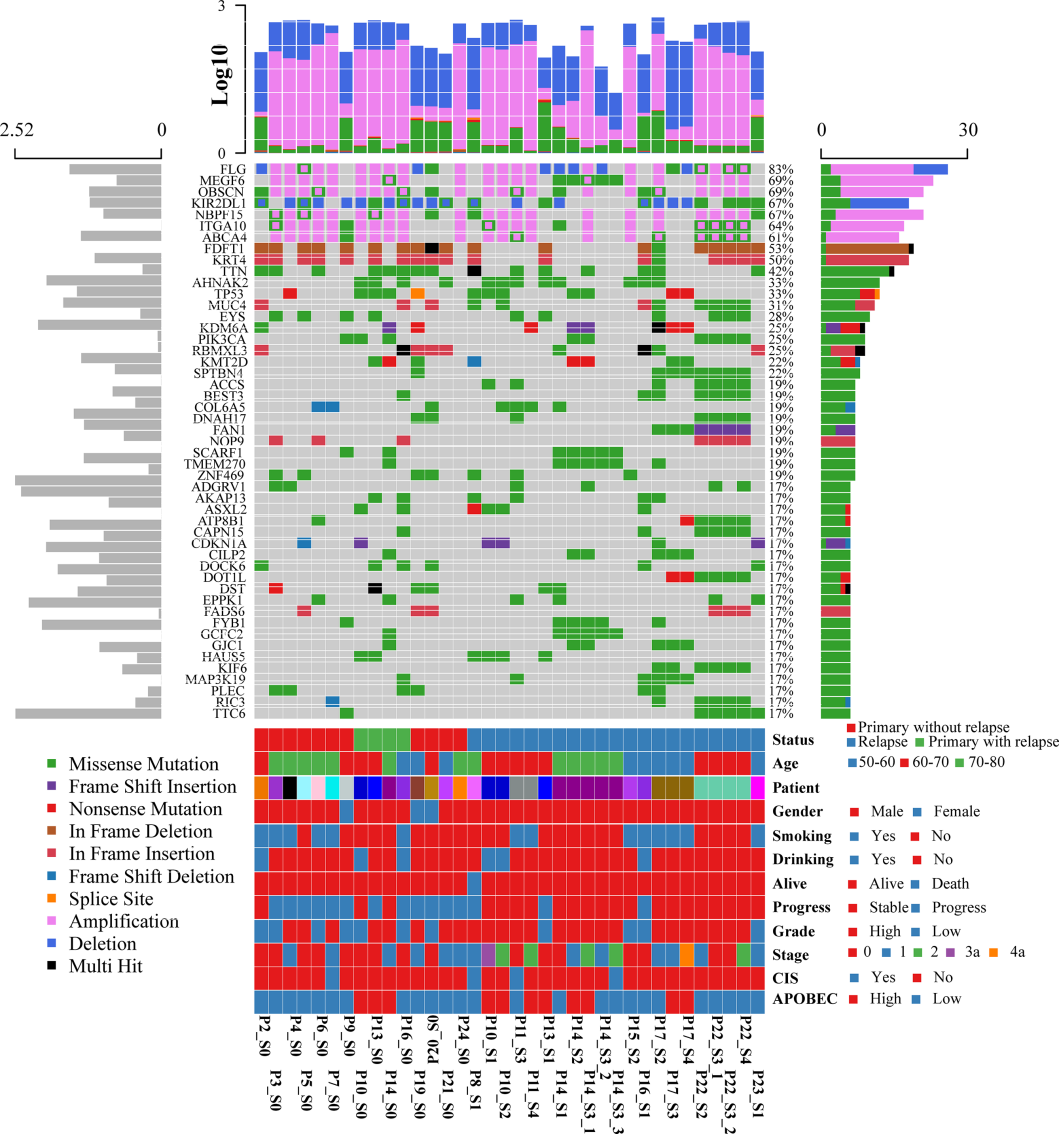
Oncoplot displaying the somatic landscape and clinical information of the BCG treatment cohort. Genes are ordered by their mutation frequency, and samples are ordered according to disease histology as indicated by the annotation bar (bottom). The bottom annotation bar contains status (red for primary tumor without relapse, green for primary tumor with relapse, and blue for relapsed tumor), age, patient, gender, smoking, drinking, alive, progress, grade, stage, CIS, and APOBEC-enriched (from top to bottom). The left side bar plot shows log10 transformed average of transcripts per million reads (TPM) estimated by StringTie2. The top side bar plot shows the log10 transformed of tumor mutation burden, including SNV and CNV. The sample names on the bottom contain three parts: “Patient-ID_Different-Time-Point(_Different-Areas).” The first two parts are necessary. First is the patient ID, which is unique. The second part is for different time points: _c stands for normal tissue adjacent to the tumor; s0 stands for primary tumor; s1, s2, s3, s4 stand for different time points of the relapsed tumors. The third part is for different tumors if there are more than one tumor during cystoscopy.

To systematically explore the genomic heterogeneity during BCG treatment, we performed WES in 36 prospectively collected, transurethral resection (TUR) tumor samples from NMIBC patients, consisting of 25 high- and 11 low-grade tumors and matched normal samples (**Table S2**). RNA sequencing was also performed in 19 related samples (P12, P18, and P23 failed to extract RNA, P1 and P11 failed to build sequence library). The somatic mutations of primary and recurrent tumors were assessed by WES at the median depth of 96 (mean depth: 91; range: 44–126; **Table S2**) and without cross-individual contamination (**Figure S2**). In our cohort, the most frequently altered genes were *FDFT1* (53%), *KRT4* (50%), *TP53* (33%), *TTN* (23%), and *AHNAK2* (21%) in the primary tumors (**Figure 1** and **Table S3**). Mutations in TP53, which were found in 76% of muscle-invasive bladder cancer (MIBC) (9) and 21% of NMIBC (10), were validated by Sanger sequencing. The mutational spectrum of bladder cancer from Chinese and Western patients exhibited notable differences. Mutations of AHNAK2 and TTN were mainly from the Chinese cohort, while mutations of TERT and STAG2 were mainly from the Western cohort (**Figure S3A**). The mutation frequencies of several genes (such as *GFRA2* and *DOK2*) were significantly different between high- and low-grade tumors (**Table S4**). *DOK2* plays an important role in multiple immune response signaling pathway, such as IL-23 and IL-4 signaling pathways (11). We then assessed the mutation frequency of NMIBC-associated genes by MutSigCV in all samples and identified 19 significantly mutated genes (**Table S5**). Mutations in *CDKN1A*, a known bladder cancer driver gene, were the most significant event occurring in 17% of tumors (12). Based on the annotation of gene ontology, membrane proteins including *EXOSC10*, *DGAT2L6*, *CRLS1*, and *DRD5* were significantly enriched. Of all the mutated genes that occurred in at least five tumors in our cohort, only *AKAP13* mutations were significantly associated with an increased risk of recurrence after BCG treatment (hazard ratio = 7.6, 95% confidence interval: 1.9–29, *p* = 0.0035, adjusted *p* = 0.059; **Table S6** and **Figure S3B**). According to the result of GISTIC2.0, there were remarkable differences of chromosome aberrations between relapsed and primary tumors (**Figure S4**). For relapsed tumors, we detected the deletion of CDKN2A (9p21.3, *q* = 1.24 × 10^−7^) and the amplification of E2F3 (6p22.3, *q* = 7.25 × 10^−5^), PPARG (3p25.2, *q* = 0.003), and FGFR3 (4p16.3, *q* = 0.014), consistent with previous studies (12, 13). We compared the chromosome instability (CIN) score between different groups (**Figure S5**). Relapsed tumors had a significantly higher CIN score than primary tumors (Wilcoxon signed-rank test, *p* = 0.0078) when we calculated the CIN score according to a recent study (14).

### Mutational Signatures of Relapsed Tumors and Matched Primary Tumors

**Figure 2:**
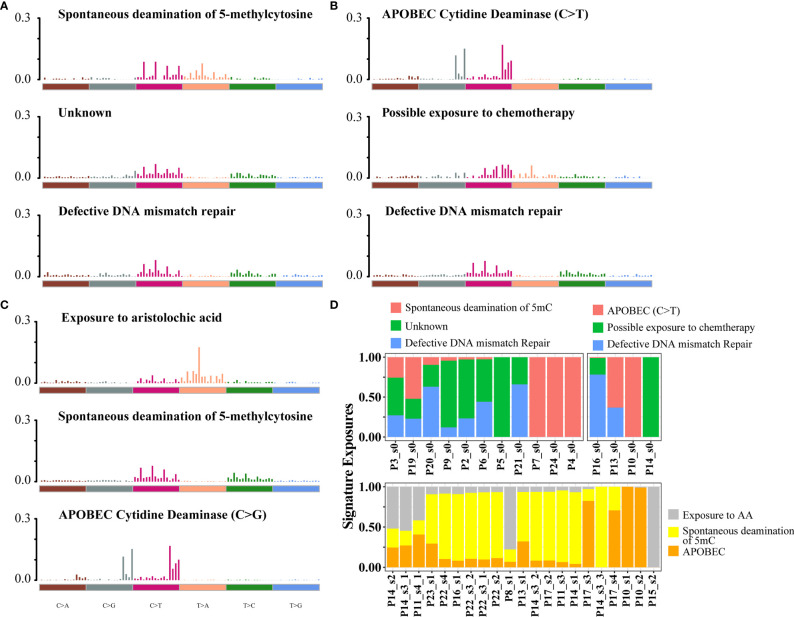
Mutational signatures identified in primary and relapsed samples, respectively. **(A–C)** Mutational signatures identified from primary tumor without relapse, primary tumor with relapse **(B)** and relapsed **(C)** samples. The *y*-axis indicates the exposure of 96 trinucleotide motifs to overall signature. The plot title indicates the best match against validated COSMIC signatures and cosine similarity value along with the proposed etiology. **(D)** The relative contribution of mutational signature catalog was extracted by NMF, and each signature contribution was estimated for each patient. Different colors stand for the corresponding mutational signatures. The *x*-axis are the different samples.

To assess mutational signatures at different stages of disease evolution, we used Bayesian non-negative matrix factorization (NMF) to identify two mutation signatures in the primary tumors and three mutation signatures in the relapsed tumors (**Figures 2A, C****)** (15). Spontaneous deamination of 5-methylcytosine (5mc), which was reported to promote pathogenesis of bladder cancer through stabilizing mRNAs (16, 17), was identified both in the primary and relapsed samples. Nonetheless, there were some shifts in the relative contributions of mutational processes over time. APOBEC-mediated mutagenesis plays a major role in MIBC (9). The signature of C>T transitions at CpG dinucleotides with APOBEC3A/APOBEC3B (signature 2 by the COSMIC database, cosine-similarity: 0.805) contributed to a relatively higher proportion of mutations in primary tumors. Mutations attributed to the activity of APOBEC enzymes, characterized by C>G variant with APOBEC1 in a TpC context (signatures 13, cosine-similarity: 0.808), were more prominent in the relapsed tumors (72.4%, **Figure 2B**). There was a trend of enrichment of APOBEC-mediated mutations in relapsed compared with primary tumors, although the difference did not reach statistical significance (*p* = 0.29, Fisher’s exact test, **Figure S6**). We then explored the relationship between APOBEC signatures and specific gene mutations and found that APOBEC-enriched tumors were more likely to have mutations in DNA damage response genes (*TP53*), chromatin-related genes (*ARID1A*, *KMT2D*, *HAUS5*), and transcription factors (*STOX1*), while APOBEC-low tumors were more likely to have mutations in *FDFT1* (**Figure S7**). Another interesting finding was that in the relapsed tumors, a mutation signature associated with exposure to aristolochic acid (AA, 16.2%; cosine-similarity: 0.955; **Figure 2D**), consisting of A>T transversion, with the highest proportion of 5′-TTG-3′>5′-TAG-3′ mutation, was highly enriched (**Figure S8A**). We also observed that the percentage of splice donor variant (T>A) in our cohort exhibited more than 10-fold overrepresentation than those in the TCGA or BGI cohort (**Figure S8B**). In addition, the mutations in relapsed tumors showed a strong transcriptional strand bias (T>A, *p* < 0.01; **Figure S8C**), indicating a high activity of transcription-coupled nucleotide excision repair in the relapsed tumors to repair AA-mediated mutations.

### Mutational Burden Analysis

**Figure 3:**
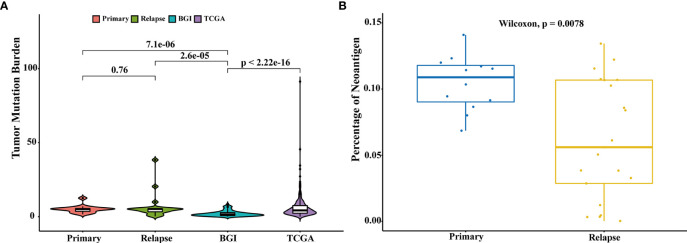
Comparison of tumor mutation burden and neoantigen load. **(A)** Comparison of the tumor mutation burden across different cohorts: primary (*n* = 15), relapsed (*n* = 21), TCGA (*n* = 413), and BGI (*n* = 99). Comparison of TMB of different groups was performed with Wilcoxon test and *p*-values were labeled. **(B)** Comparison of the percentages of neoantigens between primary and relapsed samples. Comparison of the percentages of neoantigen was performed with Wilcoxon test.

**Figure 4:**
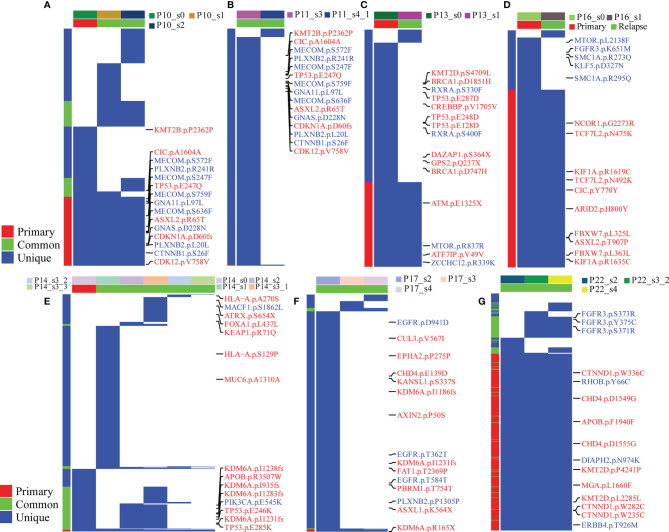
Branching evolution in BCG-treated tumor samples. The fraction of tumor cells with common, primary, and unique clonally adjusted mutations for each tumor sample is shown for all patients with at least more tumor samples per patient. Blue color stands for mutations in the corresponding genes. In the right panel of each figure, labels with red color stand for tumor suppressor genes and blue for oncogenes. In the left panel, the red color is the mutations detected in all samples, the blue color is the mutations found in more than one sample, and the green color is for the unique mutation in one sample. In the top panel, different colors stand for the group information (primary or relapsed) and samples. **(A–G)** stand for different patients: **(A)** for P10, **(B)** for P11, **(C)** for P13, **(D)** for P16, **(E)** for P14, **(F)** for P17 and **(G)** for P22.

The association between high tumor mutation burden (TMB) and favorable outcome following immune checkpoint inhibition therapy has been well documented (18, 19). The average TMB in our patient cohort was 6.8 mutations per Mb (range: 10–1,466 mutations), and we did not find a statistically significant change in the mutation burden between primary and relapsed tumors (Wilcoxon signed-rank test, *p* = 0.76, **Figure 3A**). However, there was a significant difference of TMB between NMIBC and MIBC, including the cohort of TCGA (9) (*n* = 413, Wilcoxon signed-rank test *p* < 0.01) and BGI (13) (*n* = 99, Wilcoxon signed-rank test, *p* < 0.01). Examination of specific mutations indicated that there were significantly different mutation patterns unique to primary or relapsed tumors (**Figure 4**); the average number of mutations unique to primary and relapsed tumors was 37.26 ± 15.7 and 34.50 ± 34.46, respectively. However, the primary tumors with recurrence had higher TMB than the primary tumor without recurrence (Wilcoxon signed-rank test, *p* = 0.04). On the other hand, we identified a median of 58 predicted neoantigens in the 36 tumors (range: 1–183), which were verified by RNA-sequencing data (**Table S7**). A summary of the Venn diagrams revealed substantial variations among the time serial tumor samples from the same patient (**Figure S9**). Notably, the predicted expressed neoantigen burden was not correlated with TMB, and there was a significant decrease of the percentage of neoantigen in the relapsed tumors (Wilcoxon signed-rank test, *p* = 0.0078, **Figure 3B**), which was also previously reported in a chemotherapy-resistant MIBC cohort (20). Also of interest, 31 (86.11%) tumors harbored at least one somatic mutation in genes related to DNA damage repair. However, additional analyses with MSIsensor found that all cases had microsatellite stable tumors (percentage < 10%, **Table S2**).

### Intratumor Heterogeneity and Clonal Neoantigen Analysis

**Figure 5:**
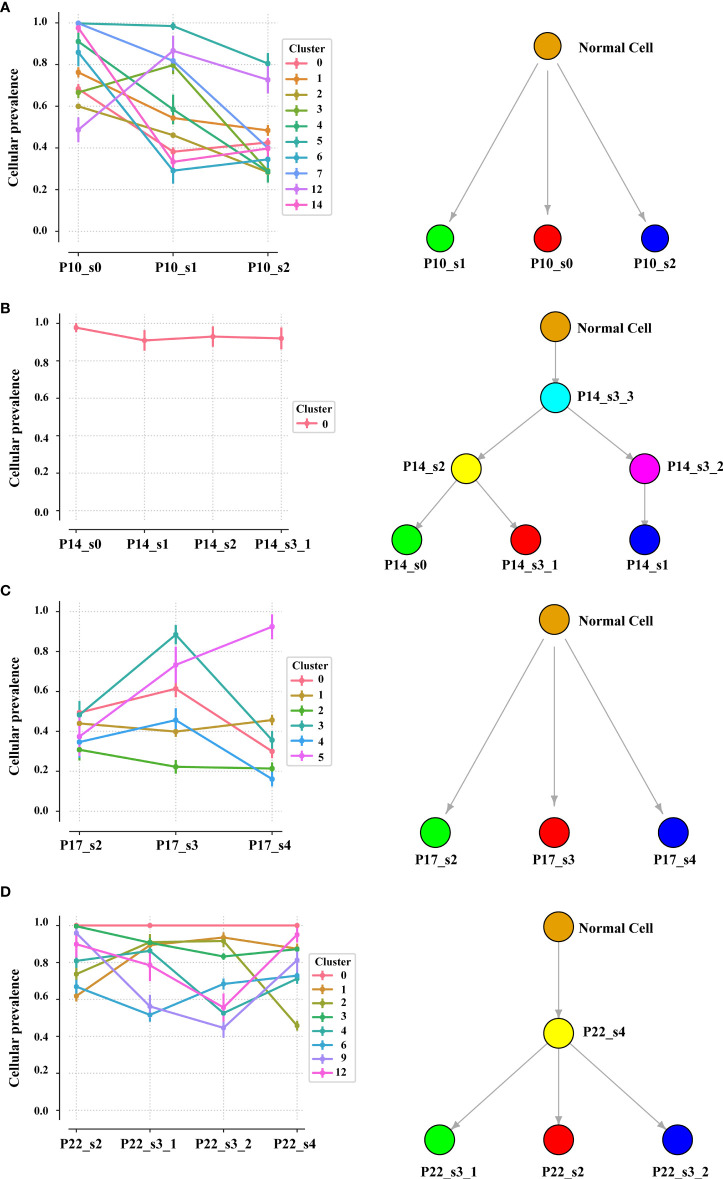
Inferred model of clonal architecture and evolution from different patients. Composition profiles generated by PyClone (left) plot the mean cellular prevalence of the variants in each cluster and phylogenetic trees from the OncoNEM algorithm (right) in the patient with at least two tumor samples. The order of the samples along the *x*-axis is temporal. The size of the marker for each mean cellular prevalence measurement is proportional to the number of variants in the cluster. Vertical lines at each point represent one standard deviation.

Intratumor heterogeneity (ITH), manifested by the distribution of clonal versus subclonal mutations, may also influence immune surveillance (21). In order to analyze the dynamic distribution of ITH, we applied PyClone to conduct a phylogenetic analysis of four sets of matched tumors from patients who had at least three tumor samples available (**Figure 5**). PyClone used copy number data and tumor purity to estimate the cellular prevalence of each variant based on its alternate allele frequency, grouping variants into clusters based on their cellular prevalence profiles across samples (**Figure S10**). Cellular prevalence plots along with inferred phylogenies for all tumors are shown in **Figure 5**. Three of four tumor samples showed evidence of subclonal structure, and the number of mutation clusters detected by PyClone varied between 6 and 15 (minimum cluster size is 2). The cellular prevalence plots provide a profile of the subclones inferred in each sample and indicate a changing subclonal composition over time. Based on the results of the neoantigen, we found C1 from P10, which contains the highest percentage of neoantigen, decreased and C5 from P17, which contains no neoantigen, increased (**Figure S11**). However, in the P22, the trend of subclonal composition was more complex. On the other hand, according to the results of OncoNEM, we found that genomic phylogenies of time-serial tumors were complex and bladder cancer had different tumor evolution models. P14 and P22 had monoclonal origin that continued to accumulate additional mutations, and each clade was therefore likely seeded by a common ancestor. P10 and P17 had multiple clonal origins that showed distinct mutational pattern. The clades of genomic phylogeny of P10 were with driver mutations: P10_s0 (PIK3CA p.M1040I) and P10_s1 (mTOR p.E1331K).

### TME as an Important Player During BCG Treatment

**Figure 6:**
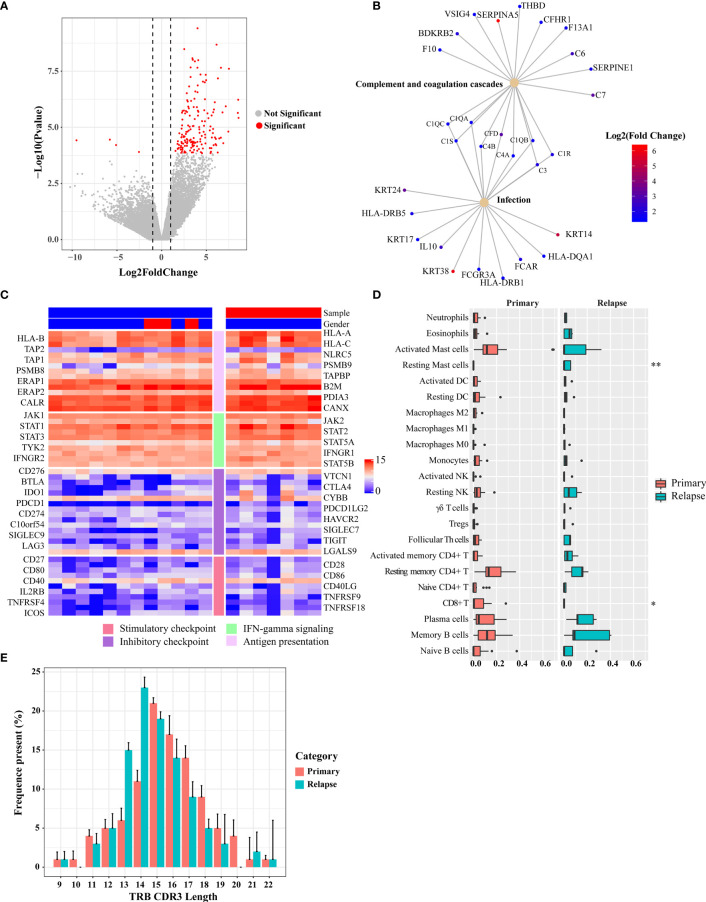
Differential gene expression between pre- and post-BCG treatment bladder cancer samples. **(A)** Volcano plot of differently expressed genes comparing primary and relapsed samples. Red symbols indicate genes that were significantly regulated. Significant genes that were upregulated or downregulated by at least two-fold are indicated. **(B)** Disease and gene association network identified by DOSE. **(C)** Expression of immune-related genes and immune checkpoint genes. The left side bar stands for different categories and the top bar for different clinical information. The color scale bar is shown at the right side of the figure. Blue indicates the lowest expression and red indicates the highest expression. **(D)** Tumor-infiltrating leukocyte levels in the primary and relapsed samples were estimated using the CIBERSORT algorithm. Comparisons were performed by employing Wilcoxon tests. *p-value < 0.1, **p-value < 0.05. **(E)** Length distribution of TRB CDR3 amino acid of the primary tumors and relapsed tumors (average of each group). The amino acid length showed a peak at 15 nt of the primary group and 14 nt of the relapsed group.

The TME plays an important role in tumor progression and metastasis and thus becomes a promising target for anticancer treatment. To identify expression changes indicative of a pharmacologic response to BCG, the baseline transcriptional programs of primary tumors and relapsed tumors (*n* = 19) were characterized using RNA sequencing, and associations with clinical response were investigated. Analysis of differentially expressed genes (DEGs) between primary and relapsed tumors identified 159 DEGs (FDR < 0.05 and fold change > 2; **Figure 6A** and **Table S8**; 156 upregulated genes and 4 downregulated genes). Notably, the upregulated genes included *S100A7*, *THBS1*, *Myosin*, and *TUBA*, which were involved in the immune and infection response. Gene set enrichment analysis with the KEGG found that multiple high-level pathways including bacterial infection and immune-related pathways such as Th1/Th2 cell differentiation and *Staphylococcus aureus* infection were significantly enriched (**Figures 6B** and **S12A**, **Table S9**). Previous studies have shown that Th1 and Th2 cytokines play a crucial role in the generation of Th1-cell responses in BCG treatment (22). For example, a key cytokine, IL-10, which was secreted by CD4^+^ Tregs and a poor prognosis biomarker during BCG therapy, was upregulated in relapsed tumors. Although none of 67 immune regulatory genes was significantly expressed (**Table S10**) (23), some T-cell exhaustion genes showed a trend toward upregulation in the relapsed tumors (**Figure S13**). Reduction of MHC-related gene expression is a frequent mechanism of immune escape. We used MHC-I score to measure the change of expression of MHC-related genes as mentioned in a previous study (24). The core genes of the MHC-I antigen presentation pathways did not show a significantly different expression (Wilcoxon signed-rank test, *p* = 0.3, **Figures 6C** and **S14B**).

Next, we explored the changes in immune subpopulations between primary and relapsed tumors using the immune-deconvolution analysis (CIBERSORT, TIMER, and quanTIseq) and observed numerous changes in immune response. The analyses of TIMER and quanTIseq show no significant difference between primary and relapsed tumors (**Figures S14C, D**). CIBERSORT-based analysis, which can deconvolve more detailed subsets of immune cells, revealed that the relapsed tumors had lower CD8^+^ T cells (Wilcoxon signed-rank test, *p* = 0.06) and higher resting mast cells (Wilcoxon signed-rank test, *p* < 0.01) compared with other subtypes (**Figure 6D**), which was also validated by RT-qPCR (**Figure S14E**). We also calculated the GSVA score with the gene signatures from a phase 2 trial of atezolizumab for metastatic bladder cancer (IMvigor210) which was used to predict PD-1 blockade treatment response (25) and found that the GSVA scores of relapsed tumors from the gene set of IMvigor210 were higher than those of primary tumors (Wilcoxon signed-rank test, *p* < 0.01; **Figure S12B**). Signatures related to CD8 T-cell activation/exhaustion were evaluated by GSVA (**Figure S12C**), and it was found that more CD8^+^ T cells differentiate into exhausted status in the relapsed tumor. This result highlights the potential clinical importance of immune checkpoint landscape in response to BCG treatment.

### Accelerated Reduction in T-Cell Receptor Repertoire Diversity of Relapsed Tumors

To assess the T-cell receptor (TCR) repertoire in the primary and relapsed tumors, we performed TCRβ sequencing by amplifying the TCRβ CDR3 region, similar to the method used in a previous study (26). One of the key determinants of TCR repertoire diversity is the length of the TCR CDR3 loop. In our study, we found CDR3 length with a peak at 15 nt for the primary tumors and at 14 nt for the relapsed tumors, respectively (**Figure 6E**). The overall composition of TCRβ CDR3 clones with different frequencies is summarized in **Figure S15A**. To show whether the overall TCR clonal expansion exhibits evenness, we summarized the unique clonotype distribution according to their abundance in each subject and found a greater proportion of TCR sequences decreased in the relapsed tumors (54%), which further suggests the narrowing of the TCR repertoire. In addition, when we calculated the Shannon diversity index (SDI) of T-cell clones, we observed a significantly lower SDI in relapsed than in primary tumors (Wilcoxon signed-rank test, *p* = 0.013, **Figure S15B**).

## Discussion

The underlying mechanisms of BCG immunotherapy are still elusive and BCG treatment failure in NMIBC patients remains an unsolved clinical challenge considering limited alternative options. WES and RNA sequence obtained at sequential time points in the same patient offered a unique opportunity to understand the mutagenic and immune pressures shaping the tumor evolution during BCG immunotherapy.

NMIBC is a heterogeneous disease with widely different outcomes. The absence of a reliable pretreatment biomarker to predict BCG outcome was attributed to the huge genetic diversity which was illustrated between our population and other NMIBC cohorts (10, 27, 28). In our study, patients harboring AKAP13 mutation showed significantly worse recurrence-free survival after receiving an induction course of BCG. AKAP13 mutations were associated with the progression of several cancers; however, no prior study examined the association between AKAP13 mutations and BCG outcomes. Compared with the study from the Memorial Sloan Kettering Cancer Center in US population, the distinct mutation pattern of NMIBC in the Chinese population explained the discrepancy in outcomes. Further work is warranted in order to clarify the potential of AKAP13 mutations as a predictive biomarker for BCG therapy.

The concept that tumors with higher TMB present more neoantigens and thus more immunogenic correspondingly (29) was questioned by the contradictions that tumors with similar TMB level exhibit a wide spectrum of immune responses and some tumors with low TMB still respond to immunotherapy well. It indicated that TMB is not the solo determinant to be considered in immunotherapy. The other factors required for T cells recognizing tumor cells, such as tumor antigenicity, tumor immunogenicity, and TME, were also viewed as promising candidates to predict clinical benefit of immunotherapy and to guide the development of rational combinations (30). The potency of the immune system to distinguish between normal and tumor cells is crucial to cancer immunotherapy and also relies on the sufficient antigenicity of tumor cells. Recent studies have demonstrated that mutated neoantigen-producing proteins could serve as effective targets for cancer immunotherapy (31). In this study, we found that the percentages of predicted neoantigens were significantly lower in the relapsed tumors, which was consistent with the finding of a previous study (2). Our finding implied that the loss of antigenicity may result in tumor immunological escape during BCG treatment. Moreover, compared with primary tumors, all the relapsed tumors had increased intratumor heterogeneity (ITH) showing more heterogeneous polyclones of tumor cells. Intratumor heterogeneity was complicated with the immunological escape in tumor relapse. In our ITH analysis, tumor subclones with a higher percentage of neoantigen were decreased in the relapsed tumors, while tumor subclones with lower neoantigen were increased. Wolf et al. suggested that tumor cells could escape from immune surveillance with high ITH because the reactive neoantigens undergo “dilution” within the tumors relative to other neoantigens (21). Grossly, the effect of tumor cells with higher ITH reduces the antitumor immunity, which manifested by reduced immune infiltration into the tumor core. Besides, we have shown that BCG treatment induces substantial changes in the TCR repertoire. The narrowing of T-cell diversity in the relapsed tumors may be due to the expansion of T-cell clones specific for antigens associated with tumor cells.

We also observed mutation signature shifts during BCG treatment: mutational signatures in primary tumors (APOBEC3A/APOBEC3B) shifted to novel mutation signatures (APOBEC1 and exposure to AAs, which was influenced by both the progression of disease natural history and the combined effects of therapies and BCG immunoediting). Chinese herbal medicines containing AAs were reported as a dose-dependent carcinogen in many cancers, including bladder cancer (32) and renal cell carcinoma (33). In Asia, cancer treatment using traditional Chinese medicine has a long history, and AA-containing herbal remedies are still applied in many occasions. Although the total number of enrolled patients in this study was insufficient for definitive conclusions, our finding implied that AA exposure may promote the recurrence of bladder cancer. Here, we confirmed the AA exposure history of the patients according to the questionnaire but unable to quantify the dosage. However, based on the analysis of the mutational signature, the relapsed tumors exhibited a genome-wide excess of T>A transversions and splice donor (T>A) mutations, as well as an enrichment of T>A on the non-transcribed strand. This implied that AA exposure may promote the recurrence of bladder cancer.

The tumor microenvironment, another key factor during immunotherapy, can either dampen or enhance antitumor responses. In our cohort, we found that immune inhibitors, such as CD276 and PD-1, were highly upregulated during BCG treatment. BCG is produced from attenuated live bovine tuberculosis bacterium and can activate innate immune responses mediated by multiple cytokines such as IL-2 and interferon-gamma (INF-γ). IFN-γ can further induce the expressions of immune checkpoint genes with subsequent evolution of aggressive disease phenotype. These observations were also suggestive of the existence of counter-regulatory mechanisms in cancer cells that may eventually modulate the response to BCG treatment. Previous studies have also reported that PD-L1 expression in both tumor cells and T-cell population in the tumor microenvironment was a predictive factor of BCG response in NMIBC (34). These data suggested that the combination of immune checkpoint inhibitor and BCG treatment may be a feasible strategy for the pharmacological intervention of NMIBC. On the other hand, the tumor microenvironment is also composed of various innate immune cells, such as mast cells (35). The role of mast cells in tumor progression remains controversial (36–38). It has been proposed that the recruited mast cells in the tumor microenvironment promote bladder cancer metastasis and correlate with poor prognosis (37, 38). In our current cohort, there were significantly more infiltrated mast cells in the relapsed tumors than in the primary tumors, indicating that mast cells may be another contributor to BCG failure.

Our analyses identified substantial temporal heterogeneity between tumors collected at different time points from the same patient. The majority of mutations in the post-BCG tumors were not shared with primary tumors. Branching evolution was the predominant path from primary, BCG-sensitive to immune-escaped bladder cancer. Detected very early in this path, several clones contained a high percentage of neoantigens. The percentage of neoantigens was reduced in the process of treatment. Meanwhile, multicentricity provided an alternative interpretation of the phylogenetic trees in which tumors with extensive biological complexity escaped the pressure of the immune system. The overall outcome is a weakened antitumor immunity, manifested by the increase of tumor heterogeneity, dampening of tumor-infiltrating lymphocyte degranulation, and reduction of tumor antigenicity.

## Conclusion

In summary, our results demonstrated that BCG-treated NMIBC patients underwent dynamic clonal evolution throughout the progression of the tumor, with significant genetic and immune editing during BCG treatment. Our findings came along with several potential clinical implications. First of all, genomic and immune divergence before and after BCG treatment suggested that the flawed strategy in identifying clinically actionable molecular targets simply focused on primary tumor before treatment. Serial biopsies across the disease progression and treatment course were warranted to capture the gradually evolving molecular landscape of a given patient’s bladder tumor. Secondly, further study of the functional role of immune checkpoint genes and TMB in mediating BCG resistance in bladder cancer may lead to a potential strategy for reversing or preventing immune therapy resistance by targeting these pathways. Finally, despite its initial effectiveness in eliminating cancer cells, BCG is associated with unintended significant mutagenic editing of the genomic landscape of relapsed tumors. Thus, the application of BCG in cancer patients should be cautiously controlled and monitored. This study shed light on the basis of BCG therapy failure in NMIBC and facilitated the development of rational therapeutic strategies for preventing and reversing the emergence of an immune-resistant state of NMIBC.

## Materials and Methods

### Ethical Statement and Cohort Characteristics

This study was approved by the Beijing Hospital Ethics Committee (BHEC). The patients in this study provided their informed consent under a BHEC-approved protocol. Patients with recurrence of intermediate- or high-risk NMIBC according to European Association of Urology guidelines following induction/maintenance BCG were eligible. Inclusion criteria were patients with any Ta/T1 or grade 3 urothelial carcinoma and/or carcinoma *in situ* (CIS). All patients were required a presumed transurethral resection of the bladder tumor (TURBT), confirmed by negative cytology and video cystoscopy with negative biopsies from suspected areas before intravesical therapy was started. Excluded were patients with solitary tumors except tumor stage T2+, age >80 years, previous treatment with BCG, previous pelvic radiotherapy or systemic chemotherapy, partial cystectomy, or intravesical chemotherapy. BCG (Immunobladder^®^, 80 mg; Japan BCG Laboratory, Japan) was given as a 1-year schedule: six weekly induction sessions and three weekly repeated maintenance sessions at months 3, 6, and 12. Patients retained BCG in the bladder for 120 min. Intravesical therapy was started at 2 weeks after the initial TURBT. Information about exposure to AA, smoking, and drinking was obtained from self-administered questionnaires. The primary end point was recurrence-free survival (RFS): the time until first recurrence including progression to tumor stage T2+, muscle-invasive disease, distant metastases, and death due to bladder cancer. Patients were followed up for at least 24 months after initial treatment at 3-month intervals, including blood analysis, urinalysis, cytology, cystoscopy, and biopsies of suspicious areas. Patients with a tumor recurrence during the 12 months of treatment underwent presumed radical TURBT and continued treatment as planned, unless this recurrence was T2+ or muscle invasive. All experimental procedures were carried out in accordance with approved guidelines. Additional clinical information was collected from the Hospital Information System. This study was approved by the State Food and Drug Administration (https://www.sfdachina.com/) (approval number: 2012L01482). All tumor samples were obtained from patients through TURBT, and normal samples were obtained far enough from the tumor (**Table S1**).

### DNA and RNA Extraction

We extracted DNA and RNA from formalin-fixed, paraffin-embedded tissues. After hematoxylin and eosin (H&E)-stained slide review and tumor tissue selection, we manually microdissected the corresponding tissue into 20 unstained, 5-μm-thick tissue sections (10 slides for DNA sequence and 10 for RNA). We purified DNA from the sample using a QIAmp FFPE DNA kit (Qiagen, Germany). For whole-exome sequencing, a minimum of 100 ng of DNA was used for the sequence library. DNA quality was determined by Caliper BioAnalyzer 2100 Instrument (Agilent Technologies, Santa Clara, CA, USA) and was confirmed by real-time PCR before sequencing. DNA samples were submitted to WuXi NextCode for next-generation sequencing using the low-input Agilent SureSelect V6 Kit. Paired-end sequencing (2 × 150 bp reads) was performed on successful DNA libraries using an Illumina HiSeq X-Ten after whole-exome capture. For RNA sequencing, we purified RNA with RNeasy FFPE kit from FFPE slides. RNA quality was determined with DV_200_ value (DV_200_ > 30%) by Caliper BioAnalyzer 2100 Instrument. RNA samples were also submitted to WuXi NextCode for next-generation sequencing with TruSeq RNA Exome. Paired-end sequencing (2 × 150 bp reads) was performed on successful RNA libraries using the Illumina HiSeq X-Ten as mentioned above.

### Analysis of Whole-Exome Sequencing Data

Sequencing adaptors and reads of low quality were removed with Trimmomatic (39). For each sample, BWA MEM (v0.7.10) was used to align the tumor and normal FASTQ files against UCSC Build Hg19 of the human genome with default parameters. PCR duplicates were removed using MarkDuplicates algorithm from Picard (v1.47, http://picard.sourceforge.net/). Local realignment was performed using GATK (v3.8.0) around novel and known variant sites followed by GATK base quality recalibration (40). These BAMs were then locally realigned and subjected to quality-score recalibration using GATK, followed by cross-individual contamination assessment using ContEst. MuTect2 was then run on the aligned, recalibrated BAMs to generate predicted somatic single nucleotide variants (SNVs). Somatic SNVs and small somatic insertions and deletions (indels) were also defined by Strelka2. Only the variations detected by two methods were used for downstream analysis. Bam-matcher was used to facilitate normal-tumor matching and avoid sample mislabeling (41).

After somatic variants calling using MuTect2 and Strelka2, the identified variants were passed through an annotation pipeline. Functional annotation was performed by ANNOVAR (v2018-04-16), using the RefGene database (2018-07-15) (42). Non-synonymous, stop-loss, stop-gain, and splice-site SNVs (based on RefGene annotations) were considered functional. Because part of the matched normal samples were missing in this project, we apply additional methods to filter out germline SNPs. In detail, variants to be removed were found in any of the following databases: dbSNP141 (modified to remove somatic and clinical variants, with variants with the following flags excluded: SAO¼2/3, PM, CDA, TPA, MUT, and OM), 1,000 Genomes Project, and gnomAD database. Variants were whitelisted if they were contained within the Catalogue of Somatic Mutations in Cancer database (v90).

MutSigCV (version: 1.4; https://www.broadinstitute.org/cancer/cga/mutsig) was used to identify genes that were mutated more often than expected by chance given the background mutation processes. The significant gene list was obtained using a false discovery rate (FDR) cutoff of 0.05. To evaluate for microsatellite instability, all cases were additionally analyzed with MSIsensor (43). MSI scores of less than 3 were considered microsatellite stable, while MSI scores from 3 to 10 were considered indeterminate, and scores greater than 10 were considered microsatellite unstable.

### Validation of Somatic Substitutions and Indels by Sanger Sequencing

Somatic substitutions and indels of TP53 were validated by Sanger sequencing based on PCR amplification. PCR primers for somatic variants are listed in **Table S11**. PCR was performed on the Dual 96-Well GeneAmp PCR System 9700 (Thermofisher, Massachusetts, USA), and 20 ng of template DNA from each sample was used per reaction. Products were sequenced with the 3730xl DNA Analyzer (Thermofisher, Massachusetts, USA). All sequences were analyzed with Sequencing Analysis Software (Thermofisher, Massachusetts, USA).

### Mutation Analysis and APOBEC Enrichment

Analysis and visualization of mutations was performed using Maftools (44). Samples were classified into two groups: “APOBEC-high” based on APOBEC enrichment >2 and Benjamini–Hochberg FDR-corrected *p*-value <0.05 and “APOBEC-low” based on APOBEC enrichment <2 and/or Benjamini–Hochberg FDR-corrected *p*-value ≥0.05. Significantly differentially mutated genes between APOBEC-high-enrichment and APOBEC-low-enrichment groups were analyzed using Maftools and visualized with oncoplots.

### Somatic Subclonal Copy Number Assessment

We determined copy number variations using CNVkit (45), run separately on each sample with the default parameters. SciClone (46) and PyClone (47) were used for the clonality analyses. To ensure the use of high-confidence clonal markers, the following variant filters were applied with SciClone: i) a minimum alternative read depth of >5 was used, ii) indels and triallelic sites were excluded, and iii) sex chromosomes were excluded. PyClone is a hierarchical Bayesian model that infers the cellular prevalence of each variant, clustering variants based on covariance of those prevalence estimates across multiple samples of the same patient. The PyClone Markov chain Monte Carlo model was run for 10k iterations for each patient, discarding the first 1,000 as burn-in. PyClone clusters were retained for subsequent analysis and reporting if they contained two or more variants, or fewer variants if the cluster contained the variant of a driver gene or if the cluster’s cellular prevalence measurement was the highest of all clusters for more than one sample.

The Genomic Identification of Significant Targets in Cancer (GISTIC 2.0) algorithm (https://www.broadinstitute.org/cancer/cga/gistic) was used to identify significantly recurrent focal genomic regions that were gained or lost (48). GISTIC deconstructed copy number alterations into broad and focal events and applied a probabilistic framework to identify the location and significance levels of SCNA. The sequencing coverage was further normalized by a set of control plasma samples from 10 healthy individuals. The chromosomal overall copy number changes were then summarized by chromosomal instability (CIN) score,


$$\text{CIN\_Score}=\sum_{k=0}^{n}V_{k}\times L_{k}$$

where *k* is the segment from CNVkit, *n* is the segment number, *V* is the *Z*-score value of a segment, and *L* is the length of a segment in base pair. An elevated chromosomal instability was defined by CIN score greater than average (controls) + 3 ∗ stdev (controls).

### Tumor Mutation Burden and Survival Analysis

The tumor mutation burden, which is defined as the total number of somatic missense mutations present in a baseline tumor sample, was determined in patients with filtered tumor samples sufficient for whole-exome sequencing. To calculate TMB per megabase, the total number of mutations counted is divided by the size of the coding region of the targeted territory (34 Mb).

### RNA-Sequencing Data Analysis

After removing reads containing sequencing adaptors and reads of low quality using Trimmomatic (39), we aligned reads to the Hg19 human genome and Ensembl annotated genes (release 83) with STAR (v2.7.1a). Gene expression levels based on RNA-seq data were measured in TPM and FPKM with StringTie2 (v1.3.6). EdgeR was used for differential expression analysis (49). To conduct the estimation of the abundances of tumor-infiltrating lymphocyte cell types, we performed an *in-silico* deconvolution of 22 immune cell types through the CIBERSORT algorithm (50), TIMER for 6 immune cell types (51), and quanTIseq for 11 immune cell types (52). Gene set enrichment analysis refers to a computational method that identifies sets of genes that are statistically enriched for a given observable variable. In this study, we searched the Gene Ontology, KEGG [by clusterProfiler (53)], and disease-gene network [by DOSE (54)] to show the enrichment and interaction of differentially expressed genes. We also checked if, among significant pathways, there is a statistically significant difference between pre- and post-BCG therapy samples. Given a pathway *P*, Fisher’s exact test determines the probability that the number of SNVs in *P* is different when considering pre- and post-BCG samples. Gene set variation analysis (GSVA), a gene set enrichment (GSE) method, was implemented to estimate the variation in pathway activity in an unsupervised manner (55). We performed the GSVA of the primary and relapse groups using the GSVA package in R. A signature score of IMvigor210 and CD8 activation/exhaustion were computed based on a previous study (25). The abundance of CD8^+^ T cells was analyzed following the method of Mocellin et al. (56).

### HLA Typing and Neoantigen Analysis

Typing at four-digit resolution using WES data was performed by OptiType for HLA class I alleles and confirmed in selected cases by molecular HLA typing during clinical routines. VCF files containing somatic non-synonymous SNVs and indels were annotated for wild-type peptide sequence using the Wildtype plugin from Ensembl-Variant Effect Predictor. Furthermore, the downstream effects of frameshift variants on the protein sequence were determined using the Downstream plugin from Ensembl-VEP. Prediction of HLA-binding neoantigens ranging from 9 to 11 amino acids in length was accomplished using pVAC-Seq (57). Neoantigens were subsequently filtered by the predicted binding dissociation constant (*K*_D_ ≤ 500 nM) and requiring that mutant peptides have greater binding affinities than their corresponding wild-type sequences.

### TCRβ Repertoire Sequencing and Repertoire Diversity Analysis

The TCRβ CDR3 regions were amplified according to a previous study (26). Amplification and sequencing of TCRB CDR3 regions was carried out on the MiSeq platform. We used Trimmomatic to clean sequencing data and FLASh (58) to get complete TCRβ CDR3 sequences. Then, sequences were assigned to their germline VDJ counterparts using IgBlast (59) against the IMGT database (60). MiRMAP (https://github.com/mikessh/migmap) was used for the statistical analysis of T-cell clones. The Shannon diversity index reflects clonal or TCR repertoire diversity within and between populations and was used to measure the diversity of the clonotype population at each time point. The Shannon diversity index (SDI) was calculated using the formula,


$$\text{SDI}=-\sum_{k=0}^{n}p_{i}\ln p_{i}$$

where *p* is the frequency of clonotype *i* for the sample with *n* unique amino acid TCRβ sequences with only the in-frame productive TCRβ sequences being considered in the calculation. Higher scores were generated for polyclonal samples, whereas a lower score indicated clonality.

### Statistical Analysis

Statistical analysis was performed using R software v.3.5.1. Wilcoxon signed-rank tests were used for continuous variables. Cox regression modeling was used to determine the association between genomic alterations and recurrence after BCG treatment. The Kaplan–Meier method and log-rank test were used for estimations of recurrence-free survival. The statistical significance of the associations between the mutations and the molecular subtypes was assessed by Fisher’s exact test. A *p*-value of <0.05 was considered statistically significant.

## Data Availability Statement

The datasets presented in this study can be found in online repositories. The names of the repository/repositories and accession number(s) can be found below: https://www.ncbi.nlm.nih.gov/bioproject, PRJNA574742.

## Ethics Statement

The studies involving human participants were reviewed and approved by the Beijing Hospital Ethics Committee. The patients/participants provided their written informed consent to participate in this study.

## Author Contributions

FX, MC, and YZ conceived the hypothesis. FS, WZ, MT, YZ, and HL performed the experiments. FS, WZ, MT, JZ, LZ, LL, MC, JM, and FX designed and interpreted the results. FS, WZ, ML, YZ, LZ, LL, and MC wrote the manuscript.

## Funding

This work was supported by the National Natural Science Foundation of China (Grant 81902618), Beijing Bethune Charitable Foundation (Grant mnzl202009), CAMS Innovation Fund for Medical Sciences (2021-I2M-1-050), the National Key Research and Development Program of China (Grant 2018YFC2000505), Beijing Hope Run Special Fund of Cancer Foundation of China (LC2019A04), the Fundamental Research Funds for the Central Universities (3332019120), and the Non-profit Central Research Institute Fund of Chinese Academy of Medical Sciences (2019PT320027).

## Conflict of Interest

The authors declare that the research was conducted in the absence of any commercial or financial relationships that could be construed as a potential conflict of interest.

## Publisher’s Note

All claims expressed in this article are solely those of the authors and do not necessarily represent those of their affiliated organizations, or those of the publisher, the editors and the reviewers. Any product that may be evaluated in this article, or claim that may be made by its manufacturer, is not guaranteed or endorsed by the publisher.
