# Supplementary material for: Bacillus Calmette–Guérin Treatment Changes the Tumor Microenvironment of Non-Muscle-Invasive Bladder Cancer
Source: Front Oncol. 2022 Mar 3;12:842182. doi: 10.3389/fonc.2022.842182 (PMC8930202; doi:10.3389/fonc.2022.842182)
Supplement: Supplementary file 1 [file DataSheet_1.docx]

*Bacillus* Calmette-Guérin Treatment Changes the Tumor Microenvironment of Non-Muscle Invasive Bladder Cancer

Fei Su^1^, Ming Liu^2^, Wei Zhang^3^, Min Tang^4^, Jinsong Zhang^3^, Hexin Li^1^, Lihui Zou^5^, Lin Li^4^, Jie Ma^6,8^, Yaqun Zhang^2†^, Meng Chen^7†^, Fei Xiao^1,5†^

^1^Clinical Biobank, ^2^Department of Urology, ^3^Department of Pathology, ^4^Department of Oncology, ^5^The Key Laboratory of Geriatrics, ^6^Center for Biotherapy, Beijing Hospital, National Center of Gerontology, Institute of Geriatric Medicine, Chinese Academy of Medical Sciences, Beijing 100730, P.R. China.

^7^ National Cancer Data Center, ^8^State Key Lab of Molecular Oncology, National Cancer Center, Chinese Academy of Medical Sciences Cancer Hospital & Peking Union Medical College, Beijing, China.

^†^**Correspondence to:**

Yaqun Zhang ([zhangyaqun3365@bjhmoh.cn](mailto:zhangyaqun3365@bjhmoh.cn))

Meng Chen ([chenmeng@cicams.ac.cn](mailto:chenmeng@cicams.ac.cn))

Fei Xiao ([xiaofei3965@bjhmoh.cn](mailto:xiaofei3965@bjhmoh.cn))

## **Supplementary Figures**

**Figure S1** Forest plot displaying hazard ratios

**Figure S2** Pairwise comparisons between Bam files using Bam-matcher.

**Figure S3** Kaplan-Meier curve for recurrence after BCG Therapy for tumors with AKAP13 truncating mutations compared to AKAP13 wild-type.

**Figure S4** GISTIC2.0 analysis showing mapping of regions of significant chromosomal amplification or deletion throughout the genome among different groups.

**Figure S5** Chromosome instability analysis across all samples

**Figure S6** Number of APOBEC-high and -low samples in the primary and relapse samples

**Figure S7** Results from the APOBEC enrichment analysis in our cohort

**Figure S8** Mutational signatures analysis of different bladder cancer cohorts

**Figure S9** Venn diagram showing neoantigen distributions among time serial tumor samples from different patients.

**Figure S10** Pie plots of distribution of PyClone clusters in each patient.

**Figure S11** Percentage of neoantigens in the different PyClone clusters

**Figure S12** Results of GSEA for mutations in BCG-treated bladder cancer.

**Figure S13** Expression change of immune-related genes.

**Figure S14** Correlation cluster of different genes in the MHC-I presentation.

**Figure S15** Comparison of TCR diversity between primary and relapse tumors

Figure S1


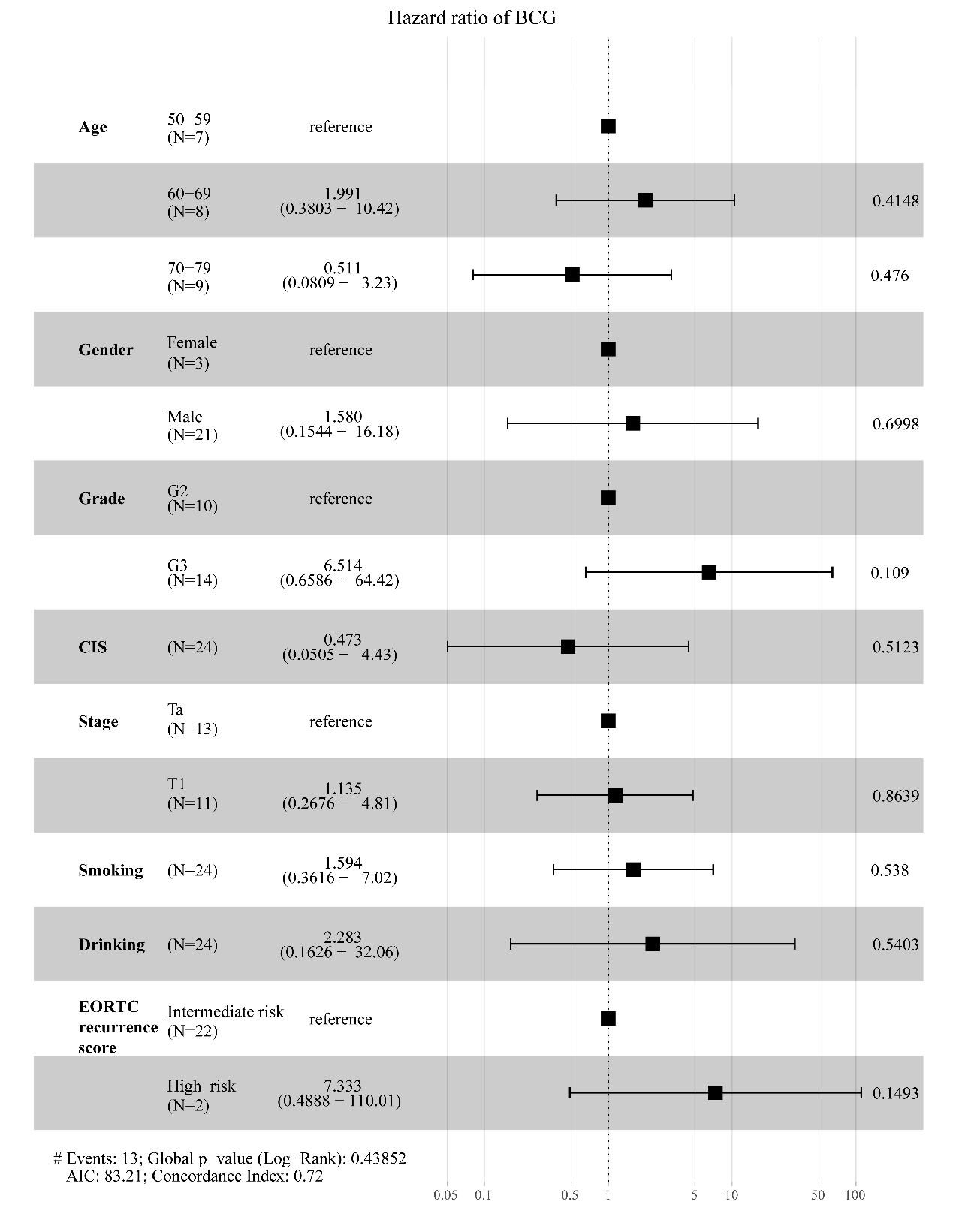


Figure S1:

Forest plot displaying hazard ratios and 95% confidence intervals (CI) for RFS with BCG therapy in patient subgroups defined by age, gender, smoking, drinking, tumor characteristics (stage, grade and CIS), and EORTC recurrence score (Intermediate risk (<9) and high risk).

Figure S2


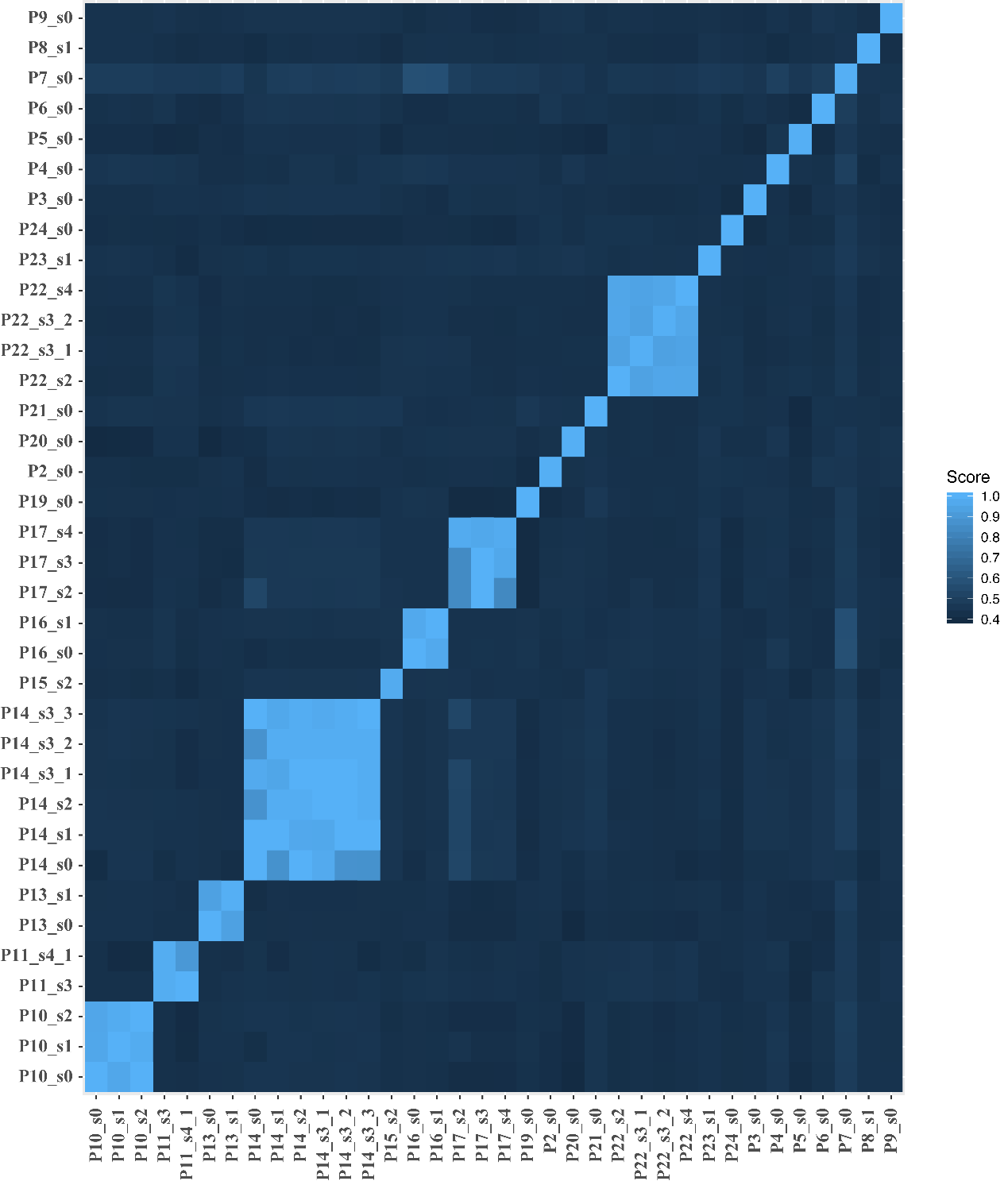


Figure legend S2: Pairwise comparisons between Bam files using Bam-matcher. Heatmap shows the results of pairwise comparison between Bam files. Different colors stand for the fraction of identical genotype calls.

Figure S3


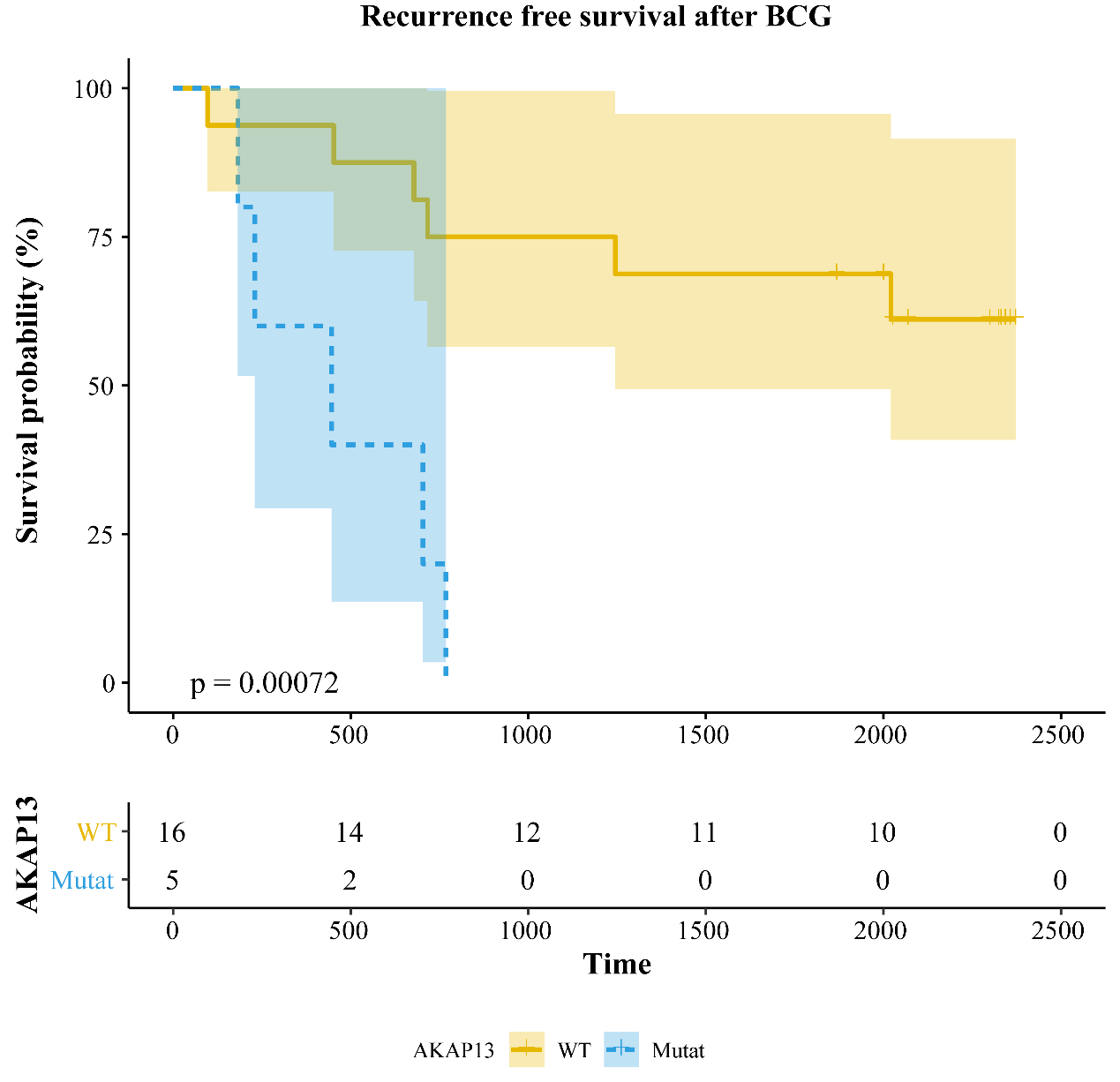


Figure Legend S3: Kaplan-Meier curve for recurrence after BCG Therapy for tumors with AKAP13 truncating mutations compared to AKAP13 wild-type. *p* was from Cox regression.

Figure S4


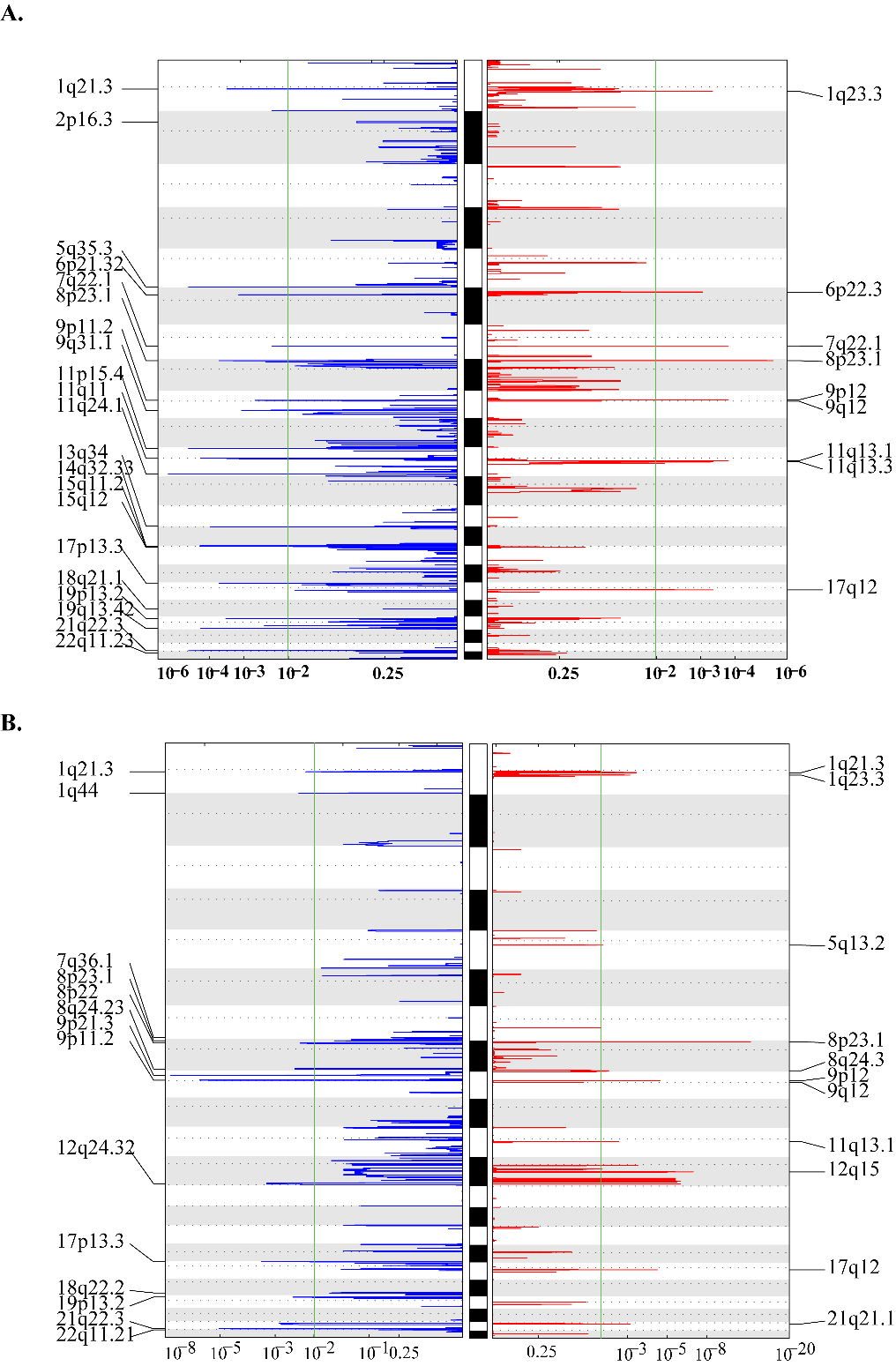


Figure Legend S4: GISTIC2.0 analysis showing mapping of regions of significant chromosomal amplification or deletion throughout the genome among different groups. Cytoband labels indicate significant calls (FDR < 0.1) A strand for primary tumors; B strand for relapse tumors.

Figure S5


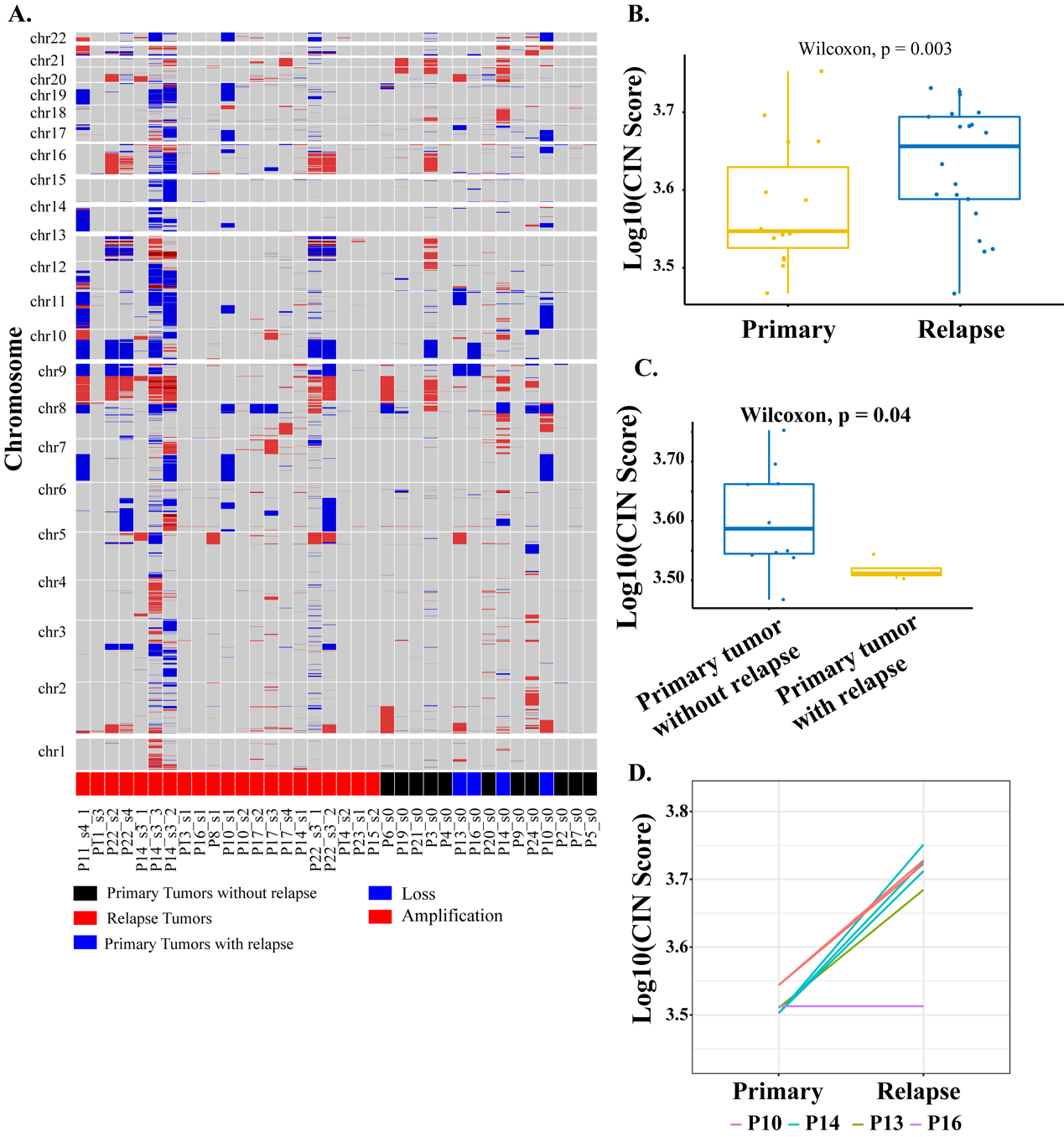


Figure Legend S5:

A. Heatmap of 36 NMIBC tumor samples by copy number alterations. Copy number gains are represented in red, and copy number losses are represented in blue. Each column corresponds to one tumor sample.

B. Comparison of chromosome instability of all primary tumors and relapse tumors

C. Comparison of chromosome instability between primary tumors with relapsed and those without relapsed

D. Comparison of chromosome instability between primary tumors and matched relapsed tumors.

Figure S6


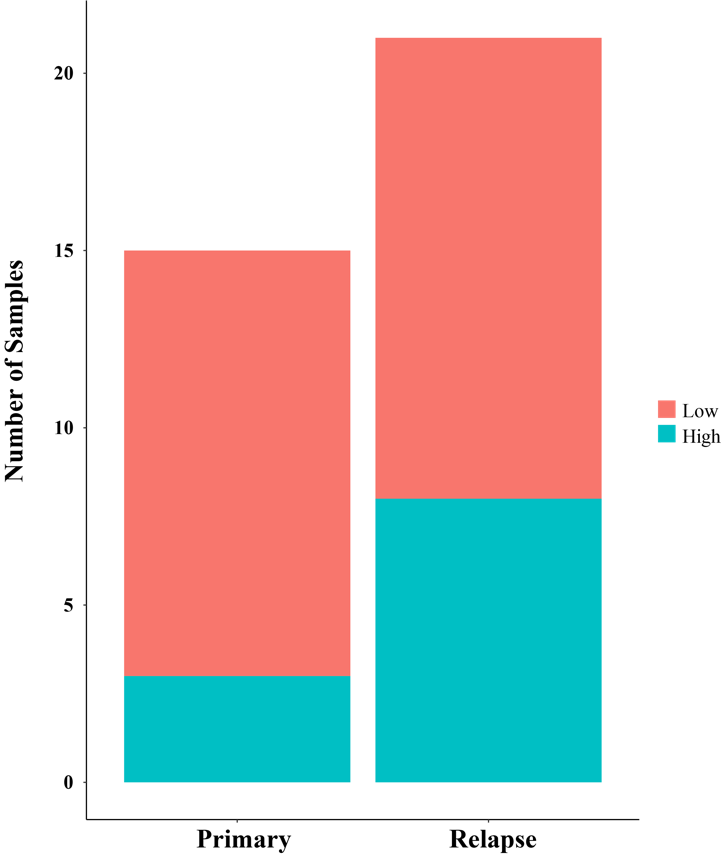


Figure S6: Number of APOBEC-high and -low samples in the primary and relapse samples.

Figure S7


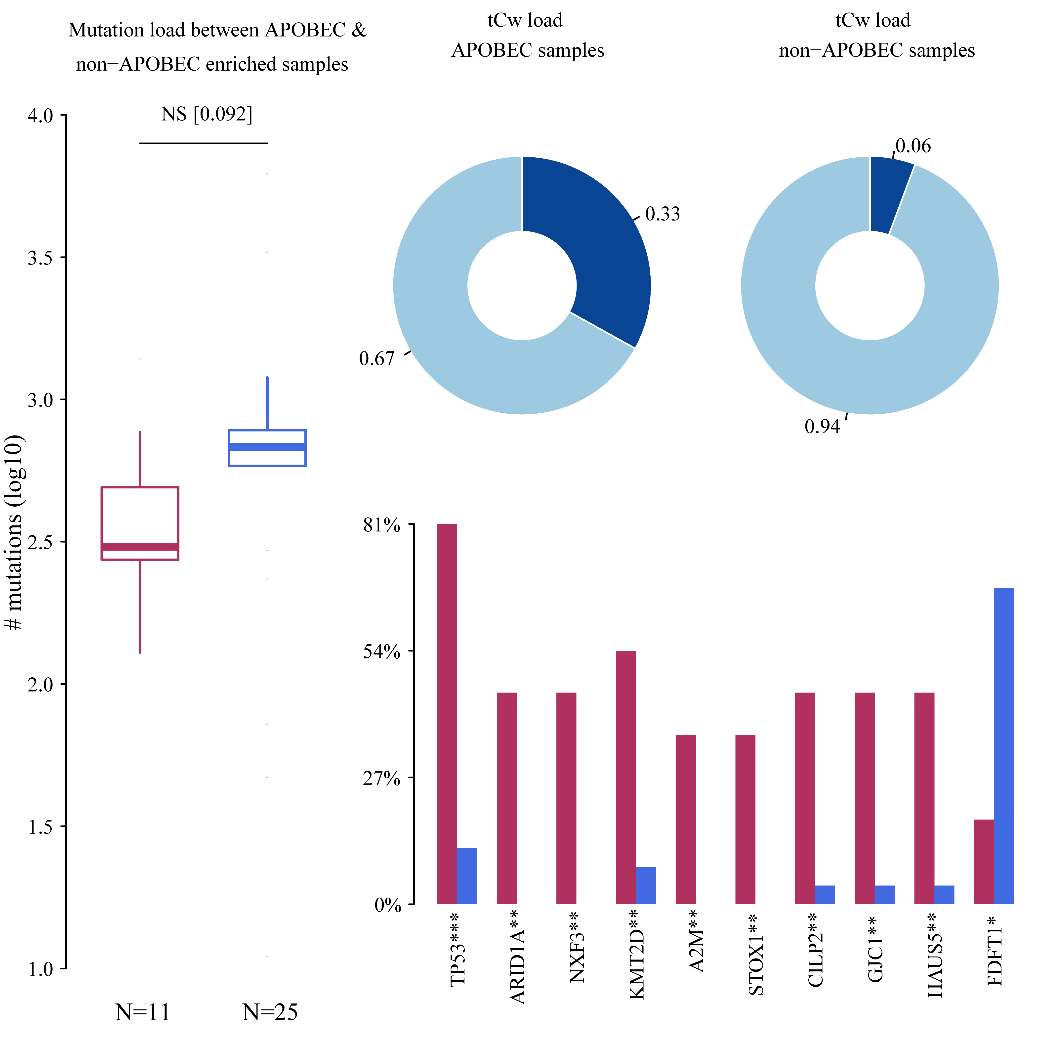


Figure S7: Results from the APOBEC enrichment analysis in our cohort. The box plot shows differences in mutation load between APOBEC-enriched and nonenriched samples: (***) Wilcoxon rank-sum test; *p* < 0.001. Donut plots display the proportion of mutations in tCw context. Bar plots show the top 10 differentially mutated genes between APOBEC-enriched and non-APOBEC-enriched samples: (***) *p* < 0.001; (**) *p* < 0.01; (*) *p* < 0.05, Fisher's exact test.

Figure S8


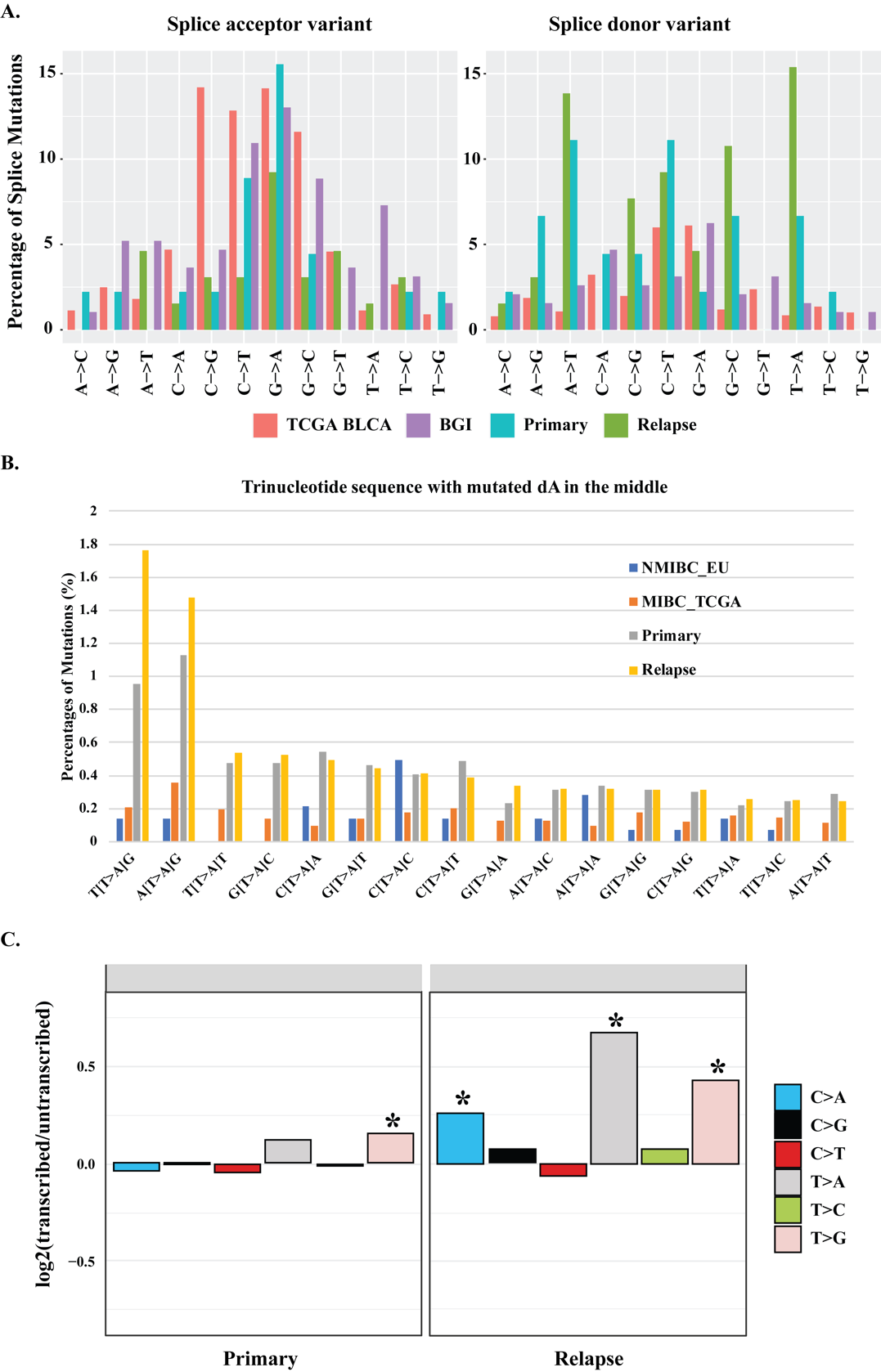


Figure S8: Mutational signatures analysis of different bladder cancer cohorts

A. Frequencies of mutations found in 5’ splice donor and 3’ splice acceptor sites in the different cohorts, including TCGA-BLCA, BGI, primary and relapse samples in this study. Mutations only in the canonical 5’ splice donor (GT) and 3’ splice acceptor (CAG or TAG) sites were counted.

B. Frequencies of T>A mutations within each observed trinucleotide sequence in the different cohorts, including TCGA-BLCA, BGI, primary and relapse samples in this study. The middle A is the mutated base (T>A) in trinucleotide sequences listed on the x axis.

C. Log2 ratio of the number of mutations on the transcribed and untranscribed strand per indicated base substitution for each tissue type. Asterisks indicate significant transcriptional strand asymmetries (p < 0.05, two-sided Poisson test).

Figure S9


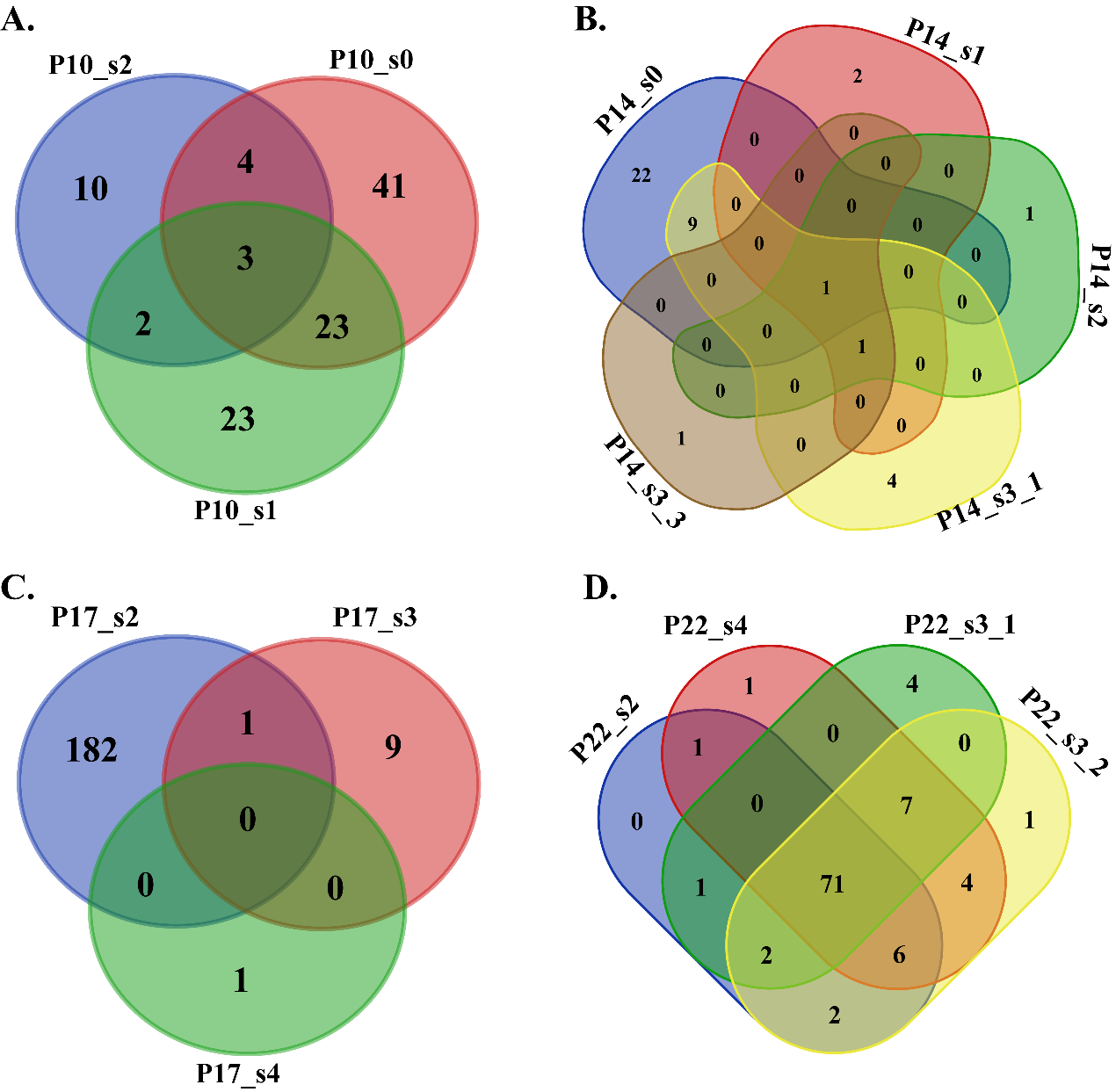


Figure S9: Venn diagram showing neoantigen distributions among time serial tumor samples from different patients. A for patient 10 (P10); B for patient 14 (P14); C for patient 17 (P17); D for patient 22 (P22).

Figure S10


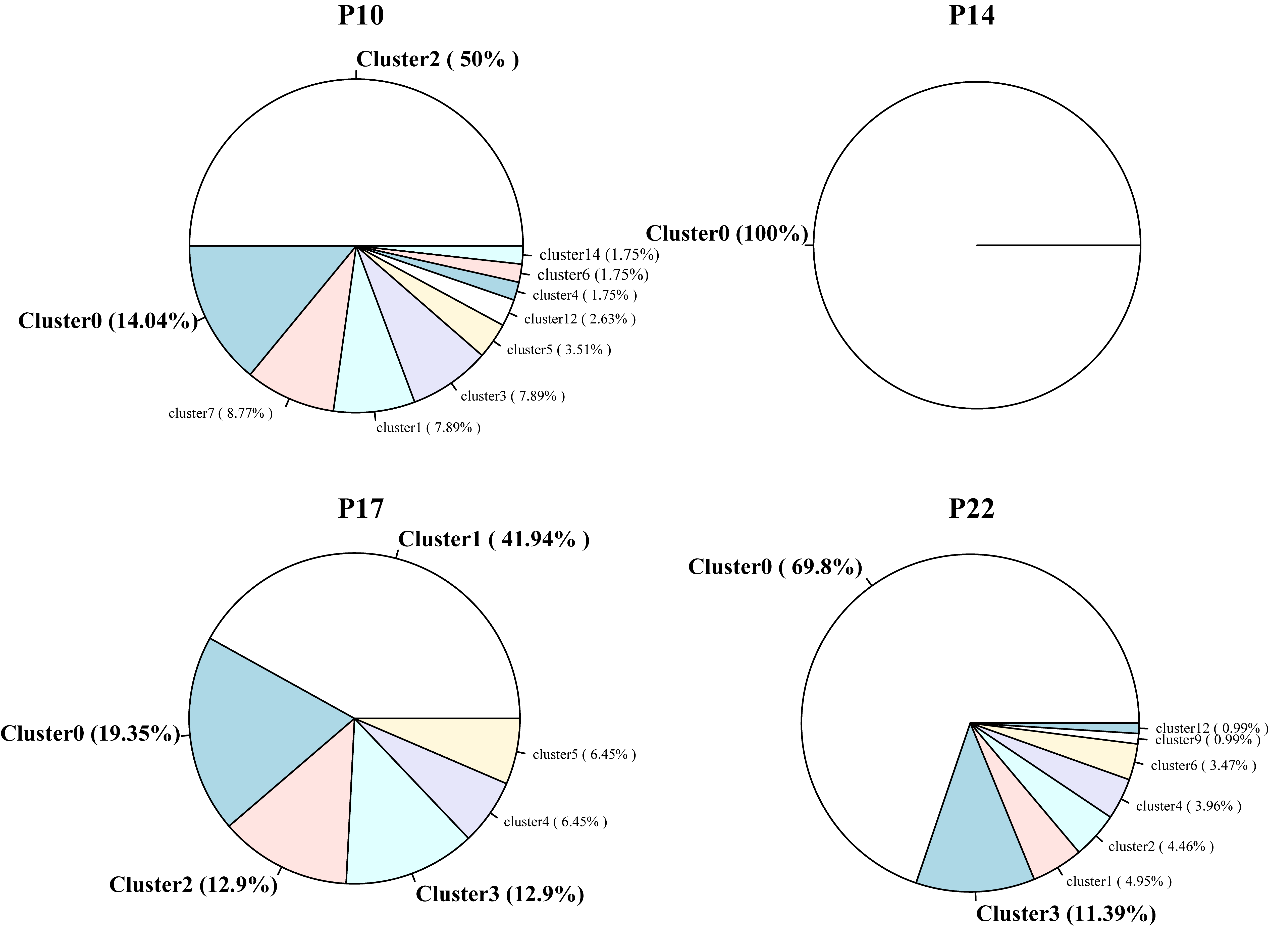


Figure S10: Pie plots of distribution of PyClone clusters in each patient.

Figure S11


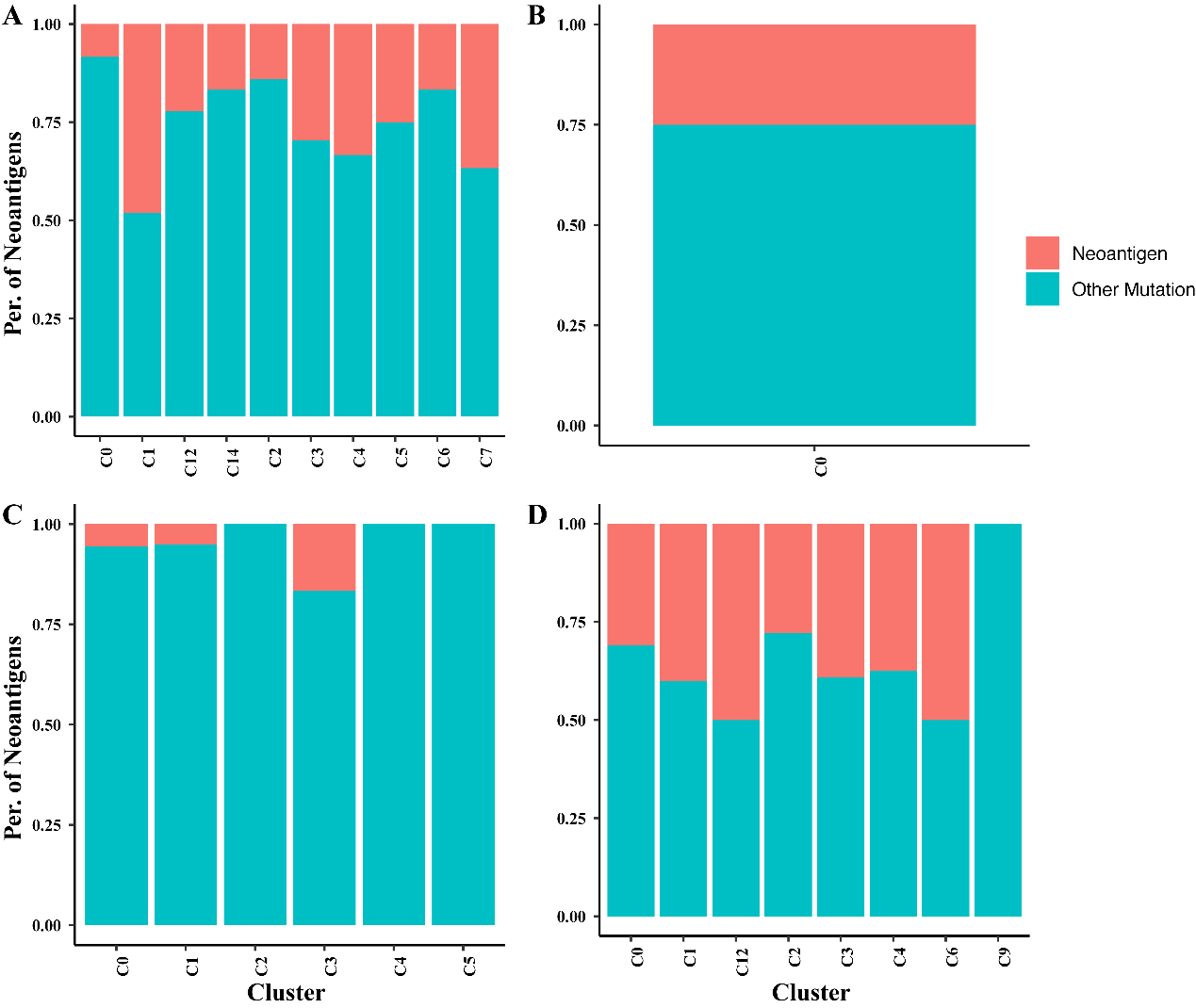


Figure S11: Percentage of neoantigens in the different PyClone clusters. A for P10; B for P14; C for P17; D for P22.

Figure S12


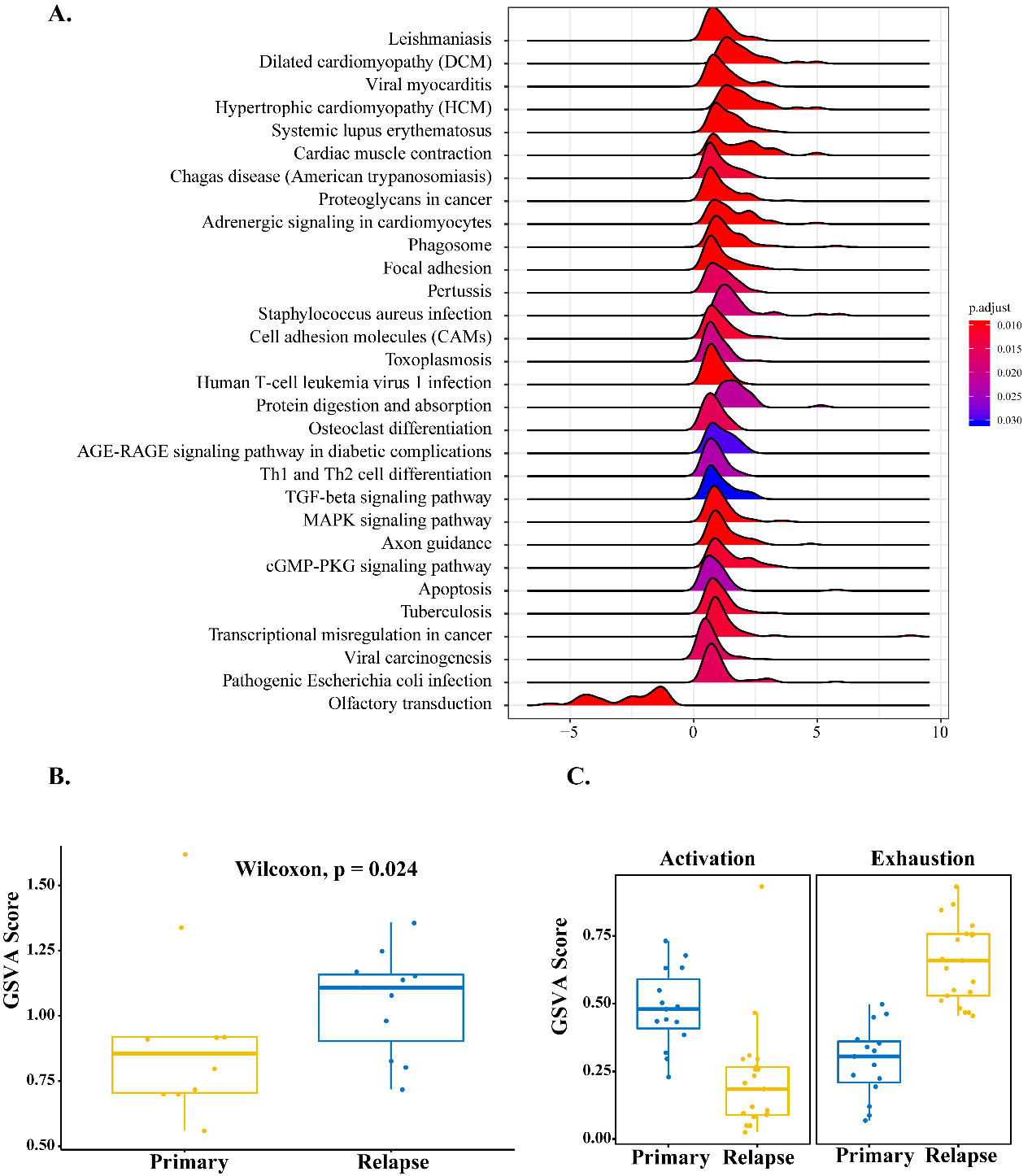


Figure S12:

A. Results of GSEA for mutations in BCG-treated bladder cancer. Each node represents one REACTOME pathway. Pathways enriched in post-chemotherapy samples are represented as red nodes. Node size corresponds to the number of genes in each pathway.

B. Comparison the GSVA score of primary and relapse tumors with IMvigor210 gene set.

C. Comparison of CD8+ T cells activation/exhaustion score between primary and relapse tumors.

Figure S13


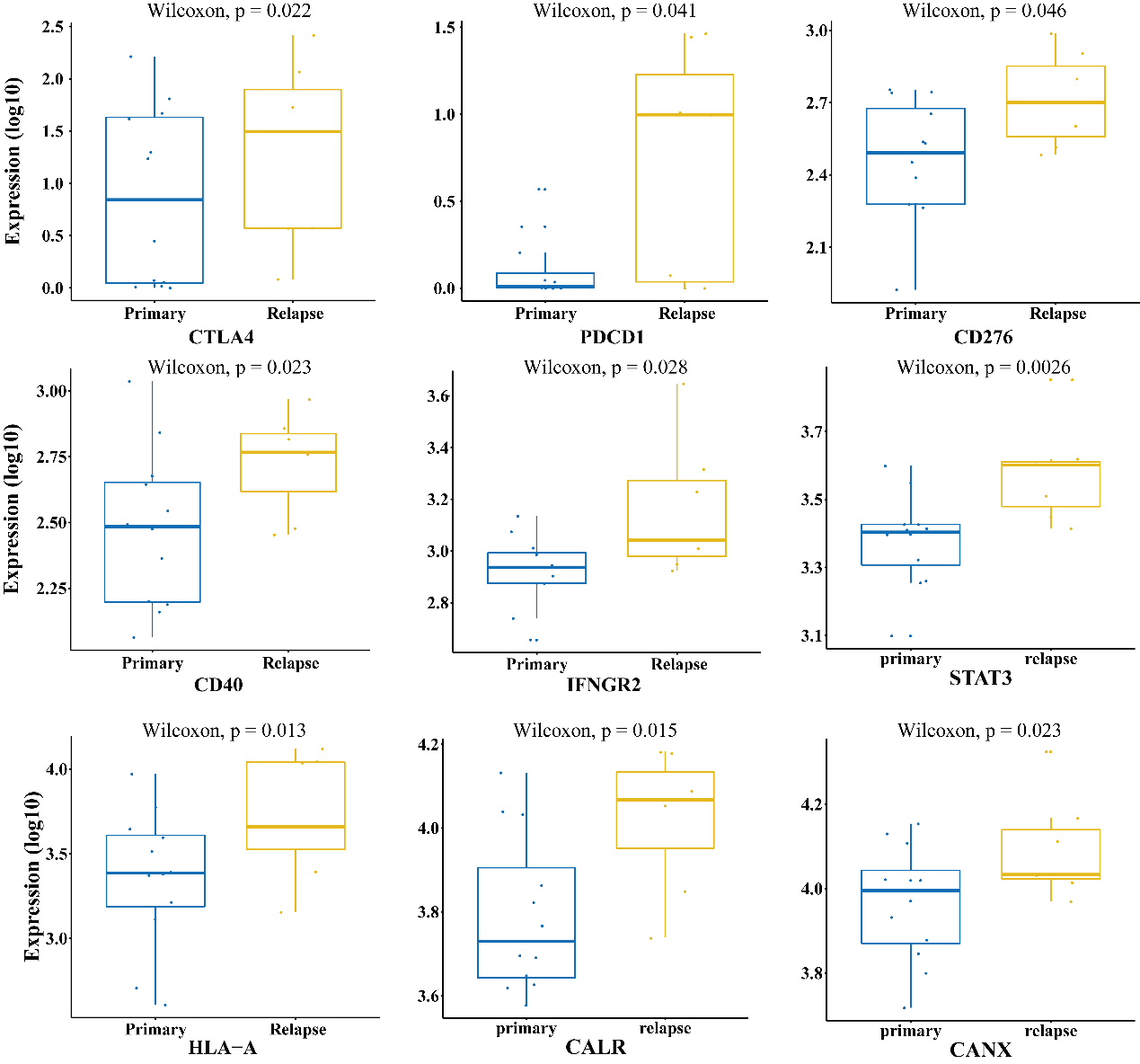


Figure S13: Expression change of selected genes, including immune inhibitor, simulator and MHC-realated genes.

Figure S14


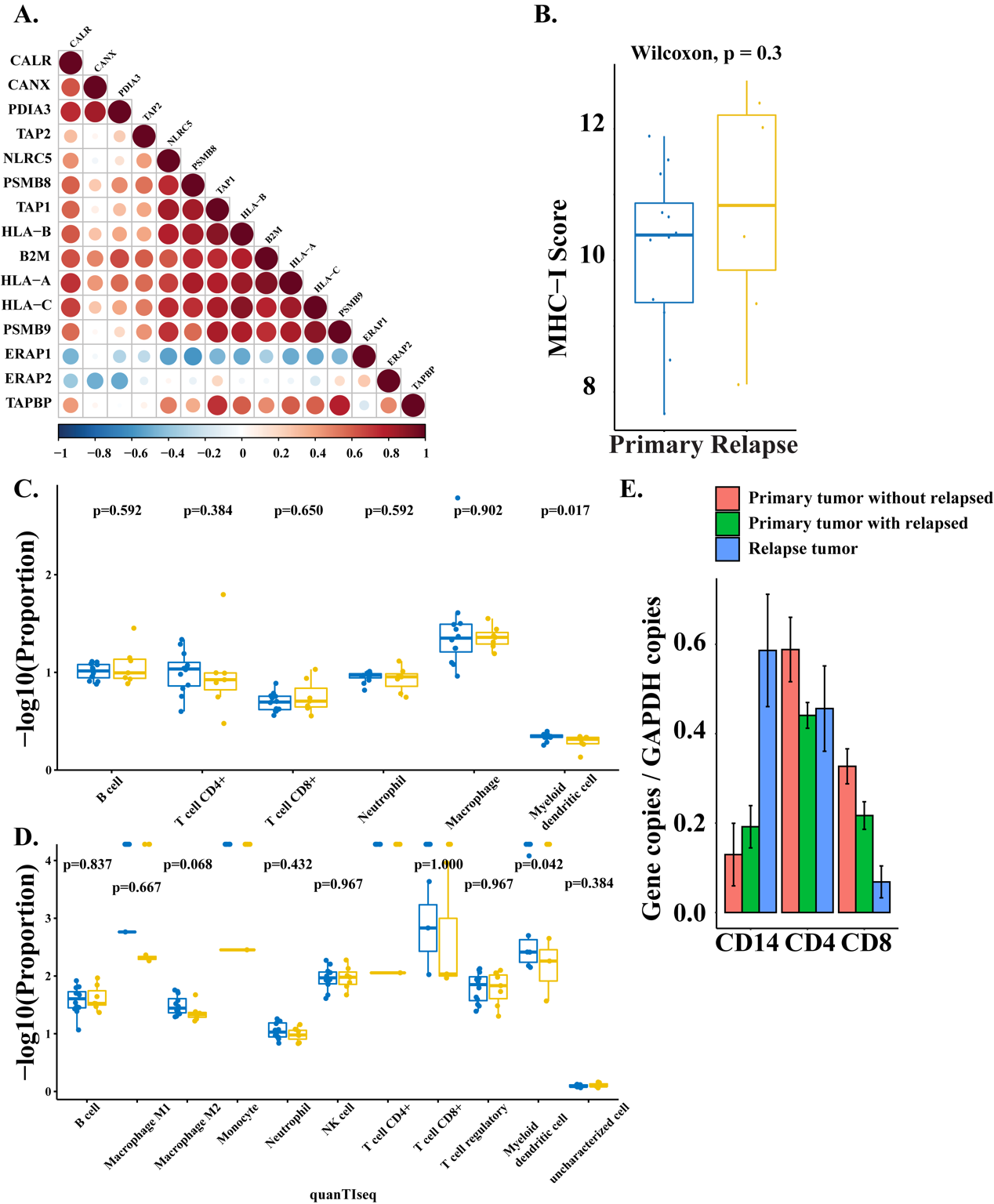


Figure S14: Correlation cluster of different genes in the MHC-I presentation.

A. Correlation matrix of MHC-I pathway genes in the ACT cohort. Genes in the red boxes displayed highest correlation and thus were termed as “core” MHC-I genes. These genes were used to create the MHC-I score.

B. Comparison of core MHC-I score between primary and relapse samples.

C and D: Results of TIMER and quanTIseq.

E. Quantitative of immune cell density measured by RT-qPCR with housekeeping genes. CD4 for helper T lymphocytes, CD8 for cytotoxic T lymphocytes, CD14 for macrophages.

Figure S15


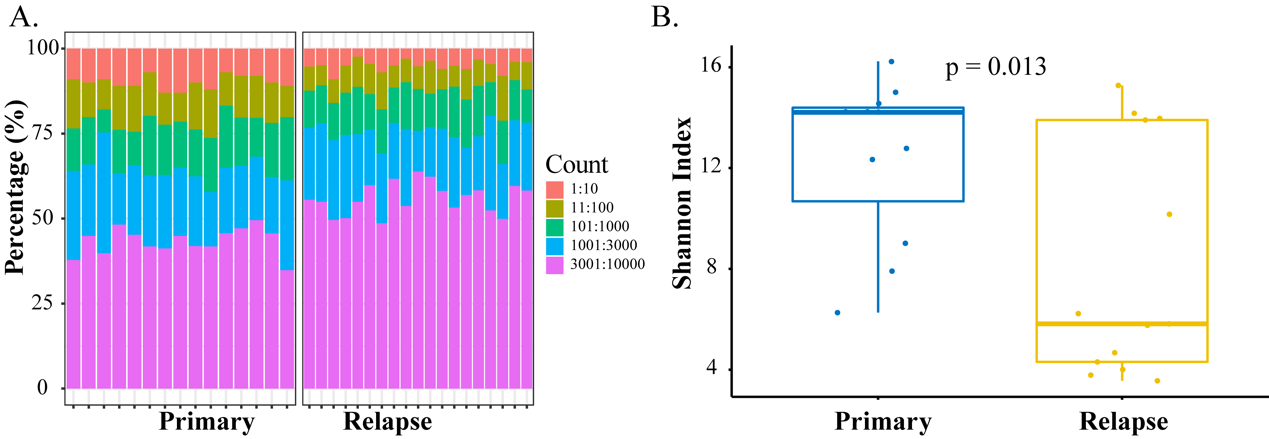


Figure S15:

A. The distributions of TCR diversity of each sample

B. Comparison of TCR diversity between the primary tumors and relapse tumors
